# Cancer-Associated Fibroblasts and Squamous Epithelial Cells Constitute a Unique Microenvironment in a Mouse Model of Inflammation-Induced Colon Cancer

**DOI:** 10.3389/fonc.2022.878920

**Published:** 2022-05-04

**Authors:** Paige N. Vega, Avlant Nilsson, Manu P. Kumar, Hiroaki Niitsu, Alan J. Simmons, James Ro, Jiawei Wang, Zhengyi Chen, Brian A. Joughin, Wei Li, Eliot T. McKinley, Qi Liu, Joseph T. Roland, M. Kay Washington, Robert J. Coffey, Douglas A. Lauffenburger, Ken S. Lau

**Affiliations:** ^1^ Department of Cell and Developmental Biology and Program in Developmental Biology, Vanderbilt University, Nashville, TN, United States; ^2^ Epithelial Biology Center, Vanderbilt University Medical Center, Nashville, TN, United States; ^3^ Department of Biological Engineering and Koch Institute for Integrative Cancer Research, Massachusetts Institute of Technology, Cambridge, MA, United States; ^4^ Department of Biology and Biological Engineering, Chalmers University of Technology, Gothenburg, Sweden; ^5^ Department of Medicine, Division of Gastroenterology, Hepatology and Nutrition, Vanderbilt University Medical Center, Nashville, TN, United States; ^6^ Department of Biostatistics and Center for Quantitative Sciences, Vanderbilt University Medical Center, Nashville, TN, United States; ^7^ Department of Surgery, Vanderbilt University Medical Center, Nashville, TN, United States; ^8^ Department of Pathology, Microbiology, and Immunology, Vanderbilt University Medical Center, Nashville, TN, United States

**Keywords:** cancer associated fibroblasts (CAF), colorectal cancer, tumor microenvironment, squamous cells, adaptive immunity, stem cells, inflammation

## Abstract

The tumor microenvironment plays a key role in the pathogenesis of colorectal tumors and contains various cell types including epithelial, immune, and mesenchymal cells. Characterization of the interactions between these cell types is necessary for revealing the complex nature of tumors. In this study, we used single-cell RNA-seq (scRNA-seq) to compare the tumor microenvironments between a mouse model of sporadic colorectal adenoma (Lrig1^CreERT2/+^;Apc^2lox14/+^) and a mouse model of inflammation-driven colorectal cancer induced by azoxymethane and dextran sodium sulfate (AOM/DSS). While both models develop tumors in the distal colon, we found that the two tumor types have distinct microenvironments. AOM/DSS tumors have an increased abundance of two populations of cancer-associated fibroblasts (CAFs) compared with APC tumors, and we revealed their divergent spatial association with tumor cells using multiplex immunofluorescence (MxIF) imaging. We also identified a unique squamous cell population in AOM/DSS tumors, whose origins were distinct from anal squamous epithelial cells. These cells were in higher proportions upon administration of a chemotherapy regimen of 5-Fluorouracil/Irinotecan. We used computational inference algorithms to predict cell-cell communication mediated by ligand-receptor interactions and downstream pathway activation, and identified potential mechanistic connections between CAFs and tumor cells, as well as CAFs and squamous epithelial cells. This study provides important preclinical insight into the microenvironment of two distinct models of colorectal tumors and reveals unique roles for CAFs and squamous epithelial cells in the AOM/DSS model of inflammation-driven cancer.

## Introduction

Polyp transition into colorectal cancer (CRC) can be modeled by an evolutionary selection of mutations favorable to tumor cell expansion (1–3). While the identities of these driver mutations have been revealed (4–7), there exists a myriad of factors that can influence mutational selection and tumor progression. According to Virchow's idea that cancers are wounds that do not heal, an aberrant wound healing process, where cells can maintain a regenerative or even embryonal state, drives tumor cell expansion. While aberrant cell states are eventually permanently fixed by mutations in oncogenic pathways, the initiation of these tumor-favorable states may be triggered by interaction with an abnormal tumor microenvironment (TME), the so-called sites of chronic irritation.

The role of the TME in CRC progression is well-supported by observations of increased microenvironmental complexity in CRC compared to less advanced lesions (8). More specifically, the increased abundance of cancer-associated fibroblasts (CAFs) in CRC is associated with more advanced disease, poor prognostic outcomes, and disease recurrence (9–12). Mouse models of CRC have also recapitulated this increased complexity associated with advanced lesions, which has been linked to growth factor, chemokine, and cytokine expression from CAFs (13–16). Indeed, a recent human tumor atlas study has identified “hubs” that consists of GREM1/RSPO3-expressing CAFs possibly co-occurring with stem-like cancer cells (17). However, there has been a lack of comparative studies describing changes in TME occurring right at the juncture between advanced adenoma and pre-invasive carcinoma. Here, we compare mouse models of advanced adenoma and early carcinoma to identify unique microenvironmental features that accompany this critical transition.

In the *Lrig1^CreERT2/+^;Apc^fl/+^* model, a single copy of *Apc* is recombined in *Lrig1*-expressing stem cells, and tumors, mirroring the sporadic tumorigenesis process, are initiated when the second copy of *Apc* undergoes a stochastic loss-of-heterozygosity event (18). Colonic adenomatous polyps are generated from cells with these genetic perturbations in the distal colon approximately 12 weeks after tamoxifen-induced recombination; these tumors are histologically advanced but do not progress to carcinoma (18). In contrast, distal colonic tumors induced by chemical mutagenesis *via* azoxymethane (AOM) and dextran sodium sulfate (DSS) results from β-catenin mutations in colonic crypt cells (19, 20). These inflammation-induced tumors also form approximately 12 weeks post-induction and may lead to invasive carcinomas when left to progress (21). Our goal was to utilize single-cell RNA-sequencing (scRNA-seq) coupled with multiplex immunofluorescence (MxIF) to characterize TME components and their interactions in these two mouse models of colorectal tumorigenesis that differ in their stage of progression. We found increased TME complexity in the AOM/DSS model of CRC compared to the adenoma model driven by APC loss, with increased diversity of CAFs and unique squamous epithelial cells that can be modulated by conventional chemotherapy.

## Results

### scRNA-seq Reveals the Complexity of the TME in AOM/DSS Colonic Tumorigenesis

**Figure 1:**
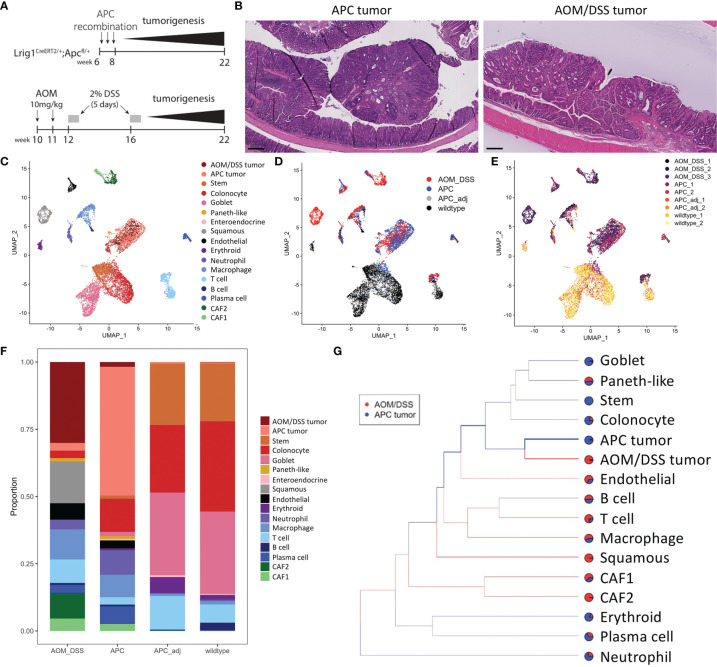
Cell type composition of wildtype, APC tumor, APC adjacent normal, and AOM/DSS tumor samples assessed by scRNA-seq. **(A)** Experimental timeline for induction of APC tumors (top) and AOM/DSS tumors (bottom). **(B)** Representative H&E images of APC tumors and AOM/DSS tumors. Scale bars = 300 µm. UMAP co-embedding of wildtype, APC tumor, APC adjacent normal, and AOM/DSS tumor scRNA-seq samples with **(C)** cell type clustering overlay, **(D)** sample type overlay, and **(E)** replicate overlay, by color. **(F)** Bar graph of cell type proportions, broken down by sample type in scRNA-seq data. **(G)** sc-Unifrac analysis of APC tumor sample cell types compared to AOM/DSS tumor sample cell types from scRNA-seq data. Colored branches indicate statistically enriched cell population structures per sample type, with black branches indicating non-significance. Pie charts indicate the proportional representation between the two sample types. Thickness of branches represent level of enrichment sc-Unifrac distance values range from 0 to 1, with 0 representing complete overlap between two datasets sc-Unifrac distance = 0.35.

We compared the cellular features between colonic tumors generated from the Lrig1^CreERT2/+^;Apc^2lox14/+^ (herein referred to as APC tumors) mice, which is a model of advanced sporadic colonic adenoma (18), to those generated by azoxymethane and dextran sodium sulfate (herein referred to as AOM/DSS tumors), which is a model of inflammation-driven CRC (19) (**Figure 1A**). We harvested tumors at 12 weeks post-induction, as documented previously to be a standard timepoint for these models and when tumor formation is routinely detected. Both sets of tumors were pedunculated and polypoid in nature, arose in the distal colon, and exhibited similar adenomatous features typical of these models (**Figure 1B**). To identify additional cellular and molecular differences between these colonic tumors, we performed inDrop scRNA-seq on multiple biological replicates (different mice) of AOM/DSS tumors, APC tumors and tumor adjacent colonic tissue, and wildtype (WT) normal colons (22). Approximately 13,000 cells across all conditions passed quality control and were co-embedded in UMAP space (**Figures 1C–E**). Density peak clustering of cell transcriptomes and subsequent automatic cell type annotation using the Mouse Cell Atlas reference revealed cell populations consistent with known biology (**Figure 1C**; **Figures S1**, **S2**) (23, 24). Normal colons from both WT and tumor-adjacent regions were virtually phenotypically identical, consisting of mostly epithelial cells with a small lymphocyte population, which we characterize below (**Figures 1C, D, F**). In contrast, tumors were composed of a much richer microenvironmental milieu (**Figures 1C, D, F**). Importantly, biological replicate data generated from different mice largely intermixed, demonstrating rigor and reproducibility (**Figure 1E**).

Examining epithelial cell types, normal colonic cells (WT and tumor adjacent) were composed of canonical stem, absorptive, and secretory cell lineages (**Figures 1C, D, F**). Tumor cells were largely distinct from normal epithelial cells. AOM/DSS tumors were devoid of goblet cells, consistent with DSS-induced inflammation that drives goblet cell loss (**Figure 1F**) (25). In addition, both tumor types displayed the emergence of metaplastic Paneth-like cells absent from the normal colon, as documented previously (26).

Within the non-epithelial compartment, tumors were enriched in neutrophils, macrophages, T cells, B cells, plasma cells, endothelial cells, and fibroblasts, compared to normal colonic samples (**Figure 1F**). We did not compare erythrocytes since their presence depends on the isolation procedure. sc-UniFrac analysis identified cell populations that were statistically enriched in AOM/DSS tumors compared to APC tumors (23). As expected, each tumor type was enriched in their respective APC or AOM/DSS tumor cell type (**Figure 1G**). Normal epithelial cells were further depleted in AOM/DSS tumors, supporting their more advanced progression stage (**Figure 1G**). Within the immune cell compartment, T cells and macrophages were enriched in AOM/DSS tumors, while plasma cells and neutrophils were enriched in APC tumors. Two populations of cancer-associated fibroblasts (CAFs), CAF1 and CAF2, were enriched in AOM/DSS tumors compared to APC tumors, with CAF2s being mostly exclusive to AOM/DSS tumors (**Figure 1G**). In addition, an unexpected cell population expressing stratified epithelial markers, such as *Krt5* and *Krt14*, was present only in AOM/DSS tumors. Together, these data highlight the expansion of the stromal compartment in AOM/DSS tumors, consistent with their more advanced state compared to APC tumors.

### CAFs and T cells in AOM/DSS Tumors Specify a Distinct Pro-Tumor TME

**Figure 2:**
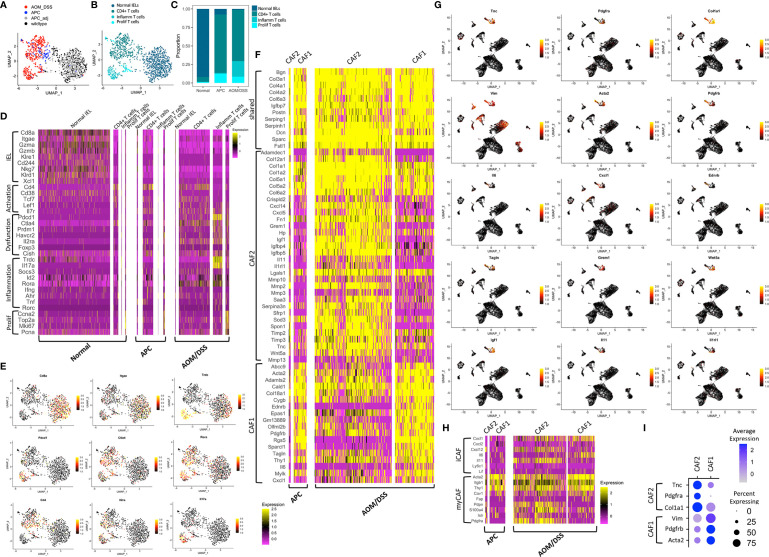
Transcriptomic differences among microenvironmental cell types in normal colonic, APC tumor, and AOM/DSS tumor samples. UMAP co-embedding of the T cell cluster from wildtype, APC tumor, APC adjacent normal, and AOM/DSS tumor scRNA-seq samples with **(A)** sample type overlay **(B)** and cell type clustering overlay, by color. **(C)** Bar graph of T cell type proportions, broken down by subcluster and sample type in scRNA-seq data. **(D)** Heatmap of select T cell subtype marker expression in scRNA-seq data within the T cell clusters, split by sample type. Columns represent single cells. **(E)** UMAPs of scRNA-seq data with gene expression overlay, indicated by the color gradient, for select T cell subtype markers. **(F)** Heatmap of DEGs in fibroblast subtypes (CAF1, CAF2) in scRNA-seq data, split by fibroblast subtype and by sample type. Columns represent single cells. **(G)** UMAPs of scRNA-seq data with gene expression overlay, indicated by the color gradient, for select fibroblast subtype markers. **(H)** Heatmap of expression of markers of iCAFs and myCAFs described in literature in CAF1 and CAF2 populations in scRNA-seq data, broken down by tumor sample. Columns represent single cells. **(I)** Dot plot showing selected markers for fibroblast subtypes, CAF1 and CAF2, where clusters include cells from both APC tumor and AOM/DSS tumor samples.

In addition to cell type annotation, we also examined molecular differences within cell types. To examine T cell subtypes, we performed sub-clustering on the T cells, population and identified four distinct T cells clusters that are condition-specific (**Figures 2A–C**). Normal colonic samples were expectedly enriched for *Cd8*+ T cells, identified as intraepithelial lymphocytes by expression of *Itgae* and *Trdc*, as observed previously (26–28) (**Figures 2D, E**). These cells express *Gzma* and *Gzmb* that play key roles in cytotoxicity, *Cd38* marking activation, and the killing effectors *Klre1*, *Cd244* (when expressed at the appropriate level), *Nkg7, Klrd1, *and *Xcl1* (29–31). In contrast, both tumor types contained low percentages of cytotoxic cells, but contained substantial percentages of *Cd4*+ T cells (**Figures 2A–C**). These cells expressed a different set of activation markers (*Tcf7, Lef1, Il7r*), and were also enriched for regulatory genes such as *Foxp3* and *Il2ra* (CD25) (**Figures 2D, E**). Tumors also contained a small population of proliferative T cells, which signifies immune activity. Comparing between tumor subtypes, AOM/DSS tumors possessed a unique population of dysfunctional inflammatory T cells (**Figures 2A–C**). These cells expressed markers of T cell exhaustion or anergy including *Prdm1, Havcr2*, and *Cish* (32, 33) (**Figures 2D, E**). Of note, *Pdcd1*, the gene encoding the immune checkpoint protein PD-1 (34), was highly upregulated in AOM/DSS tumor T cells. In contrast, *Ctla4* expression, a marker of exhausted T cells but also Tregs, was similar in APC and AOM/DSS tumor T cells (35). More importantly, AOM/DSS T cells highly expressed *Id2* downstream of TGFβ signaling, which has been shown to lead to RORα-dependent tumor-promoting inflammation, evident in this population by high *Rora* and *Il17a* expression (36) (**Figures 2D, E**). *Cd4* is downregulated but *Trdc* is characteristically upregulated, implicating that these are not Th17 cells, but are γδT cells that produce IL17 in an innate fashion to promote inflammation (37, 38). Thus, aside from immunosuppressive T cells, the TME of AOM/DSS tumor is further augmented by dysfunctional T cells that potentially induce tumor-promoting inflammation.

Within the B lymphocyte compartment, normal colonic samples were skewed toward B cells, with only a minor population of plasma cells, while both type of tumors exhibit the inverse relationship skewed towards plasma cells (**Figures 1C, D, F**). We did not notice any difference in marker genes expressed in B cells or plasma cells across normal colonic, APC tumor, and AOM/DSS samples (**Figure S3A**). Although there was an expanded myeloid compartment within both tumor types, we observed that markers of neutrophils and macrophages were largely consistent across normal colonic, APC tumor, and AOM/DSS tumor samples (**Figures 1C, D, F**; **Figure S3B**), with a few critical exceptions. Tumor associated macrophages were enriched in M2 genes, *Arg1, Il1rn*, and *Tgfb1* (**Figure S3B**). Moreover, AOM/DSS tumor associated macrophages uniquely express *Il10* and *Tnf*, implicating heightened tumor-promoting immunosuppression and inflammation. Taken together, both tumor types displayed an expansion in plasma cell, macrophage, and neutrophil compartments compared to normal colonic samples contributing to a pro-tumor TME.

There are relatively few fibroblasts in normal colonic mucosa compared to other cell types, and thus, they were not sampled in whole tissue dissociations from wildtype colonic samples. In contrast, we were able to detect one small population of CAFs, CAF1, in APC tumors, and two larger CAF populations, CAF1 and CAF2, in AOM/DSS tumors (**Figures 1C, D, F**). We used differential gene expression analysis to identify marker genes that were shared, as well as ones that distinguish the two fibroblast populations (**Figures 2F–I**). *Igfbp7*, an insulin-like growth factor binding protein and molecule involved in cell adhesion whose high expression is correlated with poor prognosis in CRC, was highly expressed in both CAF1 and CAF2 cells in APC and AOM/DSS samples (39). The CAF2 population had upregulated expression of tissue remodeling genes (*Mmp10, Mmp2, Mmp3, Mmp13, Timp2, Timp3)* and immune-related genes, including many cytokines and chemokines (*Il11, Il1rl1, Cxcl14*, and *Cxcl5*) (**Figures 2F, G**). We examined markers of recently described inflammatory CAFs (iCAFs), and found CAF2s express *Cxcl12* and *Il11* (**Figure 2H**) (40). Interestingly, iCAF markers *Il6* and *Cxcl1* were expressed at low levels in CAF1s from APC tumors, but were induced in CAF1s in AOM/DSS tumors. Similarly, we examined normal myofibroblast markers (*Acta2*, *Tagln, and Mylk*) as well as myCAF markers (*Acta2*, *Itgb1* (CD29), and *Thy1* (CD90)), and found high expression in CAF1s (**Figures 2G, H**) (31, 40). In addition to immune-related functions, we observed expression of *Grem1*, *Sfrp1*, and *Wnt5a* in the CAF2 population, all of which are reported CAF markers as well as secreted factors involved in BMP and WNT signaling, respectively (41, 42) (**Figures 2F, G**). We aimed to identify specific marker genes compatible with immunofluorescence imaging with readily available antibodies to distinguish these two fibroblast populations. *Vim* (Vimentin, VIM)*, Acta2* (smooth muscle actin alpha 2, SMA), and *Pdgfrb* (platelet-derived growth factor receptor beta, PDGFRβ) were upregulated in CAF1s, with lower expression in CAF2s, and negligible expression in all other cell types, except for *Vim* (**Figure 2I**). In contrast, CAF2s were marked by upregulation of *Tnc* (tenascin-C, TNC), *Pdgfra* (platelet-derived growth factor receptor beta, PDGFRβ), and *Col1a1* (collagen type 1 alpha 1 chain). Consistent with findings that PDGF (platelet-derived growth factor) receptors form homodimers or heterodimers when bound to various PDGF ligands, *Pdgfrb* was expressed in both CAF populations, with higher expression in CAF1s, while *Pdgfra* was exclusive to CAF2s (43). These results demonstrate that the fibroblast compartment increases in complexity in AOM/DSS tumors as compared to APC tumors, with accompanied increased expression of tissue remodeling, inflammatory, and secreted factor genes in a subset of CAFs.

Fibroblasts from the normal colon were not sampled from whole tissue dissociations due to their paucity. Consequently, we developed another dissociation strategy that enriches for stromal cells. scRNA-seq using this strategy revealed substantial de-enrichment of epithelial cells, and enrichment of immune, glial, and endothelial cells, as well as five distinct mesenchymal populations all expressing *Vim* (**Figures S3C, D**). Pericytes were identified as a distinct cell population that expresses a set of four previously defined marker genes (*Abcc9, Kcnj8, Rgs5, Cspg4*) as well as *Pdgfrb* (31, 44, 45). These cells are distinct from fibroblasts in that they express much lower levels of CAF1 and CAF2 markers *Acta2*, *Tnc*, and *Pdgfra* (45). As described by others, we identified telocytes by expression of *Foxl1*, *Pdgfra*, and *Wnt5a*, and found that they also express *Tnc* (**Figure S3D**) (46). Similarly, we identified myofibroblasts by high *Acta2* expression, along with expression of previously defined marker genes (*Tagln, Actg2, Myh11, Mylk*) (31). These results demonstrate that the normal colon also possesses fibroblastic counterparts to CAF1 (myofibroblasts) and CAF2 (telocytes), although they exist in much lower numbers.

We hypothesized that CAFs may be activated in the context of an altered tumor microenvironment. Thus, we queried expression of signaling regulator genes comparing CAFs to their normal counterparts. While several CAF2 genes were maintained in telocytes (*Cxcl12, Mmp2, Timp2*) (**Figure S3D**), many were simply not expressed in any normal colonic fibroblasts (*Mmp3, Mmp10, Mmp13, Timp3, Il1rl1, Il11, Cxcl1, Cxcl5*) (data not shown). Furthermore, while *Grem1, Sfrp*, and *Wnt5a* were co-expressed in CAF2s, only *Wnt5a* was expressed in telocytes while the other two genes were expressed in the Fibro1 population in the normal colon. *Il6* was upregulated in AOM/DSS CAF1s but it was not expressed in myofibroblasts, but instead, was expressed in Fibro1 cells. CAF2 marker *Igf1* was more highly expressed in myofibroblasts than telocytes in the normal colon, inverse to the relationship of CAF1 to CAF2. Thus, we demonstrate that while counterparts of CAFs exist in the normal colon, their expression of signaling regulators is altered in tumors.

To determine whether CAF populations observed in mice translate to human, we examined CAF1 and CAF2 gene expression within fibroblast populations from human CRC scRNA-seq datasets. Multiple populations of CAFs were identified in a set of microsatellite stable (MSS) CRC samples (**Figure S3E**) and an another set of samples consisting of broader CRC subtypes (**Figure S3F**), with two datasets showing similar results (17, 31). Distinct populations of CAF1 (*ACTA2, TAGLN, MYLK*) and CAF2 (*PDGFRA*) analogs were identified, similar to our mouse models of tumorigenesis. Normal human colonic tissues and pre-cancerous colonic lesions contain a paucity of fibroblasts consistent with our observations in the mouse (data not shown) (26). Also consistent with our mouse models, CAF2 analogs expressed a rich set of signaling regulators including *CXCL12, GREM1, IGF1, IL11*, and *WNT5A*, with a subset of cells co-expressing *WNT5A* and *GREM1* and another subset co-expressing *CXCL12* and *IGF1.* These results validate the existence of CAF subpopulations in human CRCs correspond to those found in mouse colonic tumors.

### The CAF2 Population Spatially Associates With Tumor Cells

**Figure 3:**
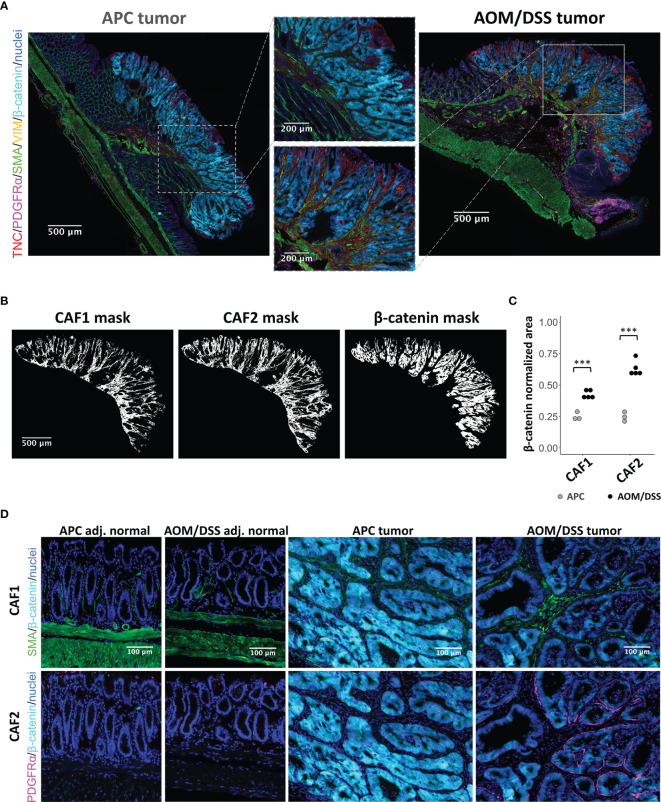
MxIF of cancer-associated fibroblasts to reveal their spatial distributions in APC and AOM/DSS tumors. **(A)** Representative stitched MxIF images of APC tumors and AOM/DSS tumors immunostained for CAF1 (SMA, VIM) and CAF2 (TNC, PDGFRα) subtypes. Tumor regions are visualized using immunostaining for β-catenin. **(B)** Representative binary images (here, AOM/DSS tumor sample) and **(C)** quantification of CAF1 and CAF2 marker expression within tumor regions of APC and AOM/DSS samples. n = 5 AOM/DSS tumors, n = 3 APC tumors *** indicates p < 0.001. **(D)** Visualization of CAF1 (SMA) and CAF2 (PDGFRα) subtype proximity to β-catenin+ tumor cells in APC and AOM/DSS samples, including tumor regions and adjacent normal regions.

We validated the presence and abundance of the CAF1 and CAF2 populations using MxIF imaging (47). As both APC and AOM/DSS tumors were induced by WNT signaling, increased cytoplasmic and nuclear staining for β-catenin was observed in tissue regions with characteristic tumor histologies (18, 48) (**Figure 3A**). Within tumor regions, we used markers informed by scRNA-seq analysis to identify CAF1 (SMA and VIM) or CAF2 (TNC and PDGFRα) populations (**Figure 2I**). By visual inspection, APC tumors were densely populated by epithelial cells, while AOM/DSS tumors had expanded inter-glandular stromal spaces occupied by non-epithelial cells (**Figure 3A**; **Figures S4**, **S5**). Consequently, AOM/DSS tumors had significantly higher levels of both fibroblast types compared to APC tumors, as quantified by normalized counts of pixels positive for markers of each CAF type (**Figures 3A–C**; **Figures S6**–**S8**). Although the CAF2s were undetected as a population in APC tumors by scRNA-seq, expression of CAF2 markers, *Tnc* and *Pdgfra,* was detected at lower levels in the CAF1 population, which is consistent with low protein expression of TNC and PDGFRα by immunostaining (**Figures 2I**, **3A–C**). As opposed to scRNA-seq where the spatial context is lost, MxIF revealed that myofibroblastic CAF1s reside in the center of the inter-glandular stromal regions, while PDGFRα+ CAF2s associate closely with the epithelial layers, lining the glands (**Figure 3D**). This result differs from the myCAF definition in pancreatic cancer, where it is the myofibroblastic CAFs that associate more closely with epithelial glands (49). These results validate our transcriptomic data and support the differential abundance of CAF1 in APC versus AOM/DSS tumors, while the CAF2 population is dramatically increased in AOM/DSS tumors and has a distinct spatial distribution.

### AOM/DSS Tumors Consist of a Unique Squamous Epithelial Component

**Figure 4:**
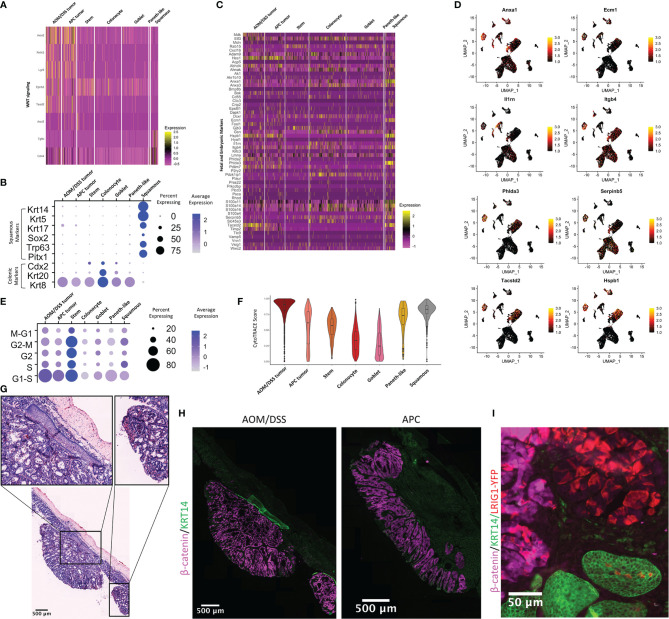
Characterization of the squamous component present in AOM/DSS, but not APC, tumors. **(A)** Heatmap of genes related to WNT signaling and colonic stem cells. Columns represent single cells, arranged by epithelial cell type clusters. **(B)** Dot plot showing colonic or squamous identity genes for epithelial cell clusters, where clusters include cells from both APC tumor and AOM/DSS tumor samples. **(C)** Heatmap of fetal and embryonic programs. Columns represent single cells, arranged by epithelial cell type clusters. **(D)** UMAPs of scRNA-seq data with expression overlay, indicated by the color gradient, of selected fetal and embryonic genes. **(E)** Dot plot showing cell cycle meta-genes for each cell cycle phase in epithelial cell clusters. **(F)** CytoTRACE scores for epithelial cell types calculated from scRNA-seq data. **(G)** Representative H&E image of multiple AOM/DSS tumors within the same animals, with a focus on squamous components (insets). **(H)** Representative MxIF image of KRT14 in APC tumors and AOM/DSS tumors. Images were representative of n = 5 AOM/DSS tumors and n = 3 APC tumors. **(I)** Observation of *Lrig1^CreERTe/+^;Rosa^YFP/+^* traced cells within a single squamous cell region of an AOM/DSS tumor.

To decipher how differences in the TME can affect tumor cells, we examined gene programs that describe signaling pathways activated in epithelial cell populations. As expected, WNT signaling genes are upregulated in tumor cells from both AOM/DSS and APC tumors when compared to normal colonic stem and normal differentiated colonic epithelial cells (**Figure 4A**). Consistent with increased stemness in tumors, the stem cell marker *Lgr5* was upregulated compared to normal cells, while colonic identity and differentiation genes such as *Cdx2* and *Krt20*, were downregulated in both tumor types (50–58)(**Figures 4A, B**). The AOM/DSS-specific squamous epithelial cell population was devoid of WNT signaling and colonic epithelial cell type markers (**Figures 4A**; **Figure S2**). While metaplasia and damage gene lists used by Chen et al., 2021 did not show enrichment in the squamous cell population, fetal and embryonic genes were uniquely upregulated in squamous cells compared to all other epithelial cell populations, including tumor cells (**Figures 4C, D**) (26). Notably, several genes related to matrix composition and immune cell recruitment were upregulated in squamous cells (*Anxa1*, *Ecm1*, *Il1rn*, *Itgb4*), as well as genes that are reported to be tumor suppressors (*Serpin5b*, *Phlda3*) (59, 60). In contrast, *Hes1*, a Notch target gene normally associated with stemness and absorptive cell differentiation, and *Phlda2*, a growth restriction gene in development and a tumor suppressor, were upregulated in APC and AOM/DSS tumor cells, but not in squamous cells (61–65). These observations are consistent with the association of tumor cells with colonic stem cell identity. Interestingly, *Tacstd2*, the gene encoding the TROP2 protein that is associated with transition to dysplasia but also expressed in some normal tissues, was highly upregulated in the squamous cell population as well as in AOM/DSS tumor cells (66, 67).

We aimed to elucidate the origin of the squamous cell population that was unique to AOM/DSS tumors. As this tumor type typically manifests in the distal colon, one possible source of this population could be re-epithelialization of damaged regions of columnar colonic epithelia by anal epithelium, as speculated by other groups (68). However, another potential origin could be the transdifferentiation of columnar to squamous epithelial cell identity (69). We first observed that keratin expression (*Krt5*+, *Krt14*+, *Krt17*+, *Krt20-*, *Krt8*-) in scRNA-seq data was consistent with a basal squamous cell identity (**Figure 4B**). *Trp63*, a master regulator of a stratified epithelial identity whose aberrant expression results in embryonic subversion, squamous cell metaplasia, and squamous cell carcinoma, was expressed exclusively in the squamous population, along with other transcription factors regulating squamous cell identity (*Sox2* and *Pitx1)* (70–73). We examined cell cycle genes, and as expected, normal stem cells are actively cycling, as are APC tumor and AOM/DSS tumor cells to a lesser extent. (**Figure 4E**). Squamous cells cycle to the same extent as tumor cells, indicative of their abnormal state. We used CytoTRACE to predict epithelial cell stemness and found that AOM/DSS tumor cells have the highest average CytoTRACE score, followed by APC tumor and then normal stem cells, consistent with higher stemness in tumor cells versus normal stem cells (**Figure 4F**) (74). Paneth-like cells also have a high score, consistent with these cells being an abnormal metaplastic cell type in tumors. Additionally, the squamous cell population’s average CytoTRACE score was similar to AOM/DSS tumor cells and above that of normal colonic epithelial stem cells, indicative of that these cells also have aberrant stemness similar to tumors. We then sought to determine whether we could locate the squamous cells spatially in tissue sections of AOM/DSS tumors, and we found examples of stratified squamous cell tissue that underlie pedunculated tumors in AOM/DSS, but not APC tumors (**Figure 4G**). Importantly, we found more than one instance where squamous components were found in multiple tumors, both distal and more proximal in the colon, within a single mouse, discounting the possibility that the squamous cells were from the anal epithelium adjacent to the tumor. Squamous components were positive for KRT14 staining, consistent with scRNA-seq results (**Figure 4H**).We then used lineage tracing from *Lrig1^CreERT2/+^;Rosa^YFP/+^* mice, as *Lrig1* is a colonic stem cell marker, to investigate whether the squamous population might arise from transdifferentiation of columnar epithelium (75). We first confirmed that *Lrig1* is not expressed in the AOM/DSS-specific squamous cell cluster, but only in the tumor cell cluster, in our scRNA-seq data (**Figure S9A**). Next, fluorescence imaging of *Lrig1^Apple/+^* and *Lrig1^CreERT2/+^;Rosa^YFP/+^* lineage traced mice revealed that LRIG1 is not expressed in normal anal squamous epithelia (**Figures S9B, C**). However, the same lineage tracing from AOM/DSS tumors resulted in YFP-positive cells in the squamous component in one instance (**Figure 4I**), suggesting a potential for rare cases of transdifferentiation. However, we cannot discount the possibility of leaky Cre recombinase activity, aberrant expression of *Lrig1* in anal epithelium in the context of AOM/DSS tumorigenesis, or other unknown factors. Moreover, squamous components were not observed in all AOM/DSS tumors, and in particular, those generated on a pure C57BL/6J background. Although we were unable to determine the origin of all squamous cells associated with tumors, we were able to validate the existence of a squamous population unique to AOM/DSS, but not APC, tumors, and whose transcriptomic signature indicates these cells have high stem potential, are actively cycling, and express several fetal and embryonic genes.

### Chemotherapeutic Treatment Results in Expansion of Squamous Cells and Depletion of Stem Features in Tumors

**Figure 5:**
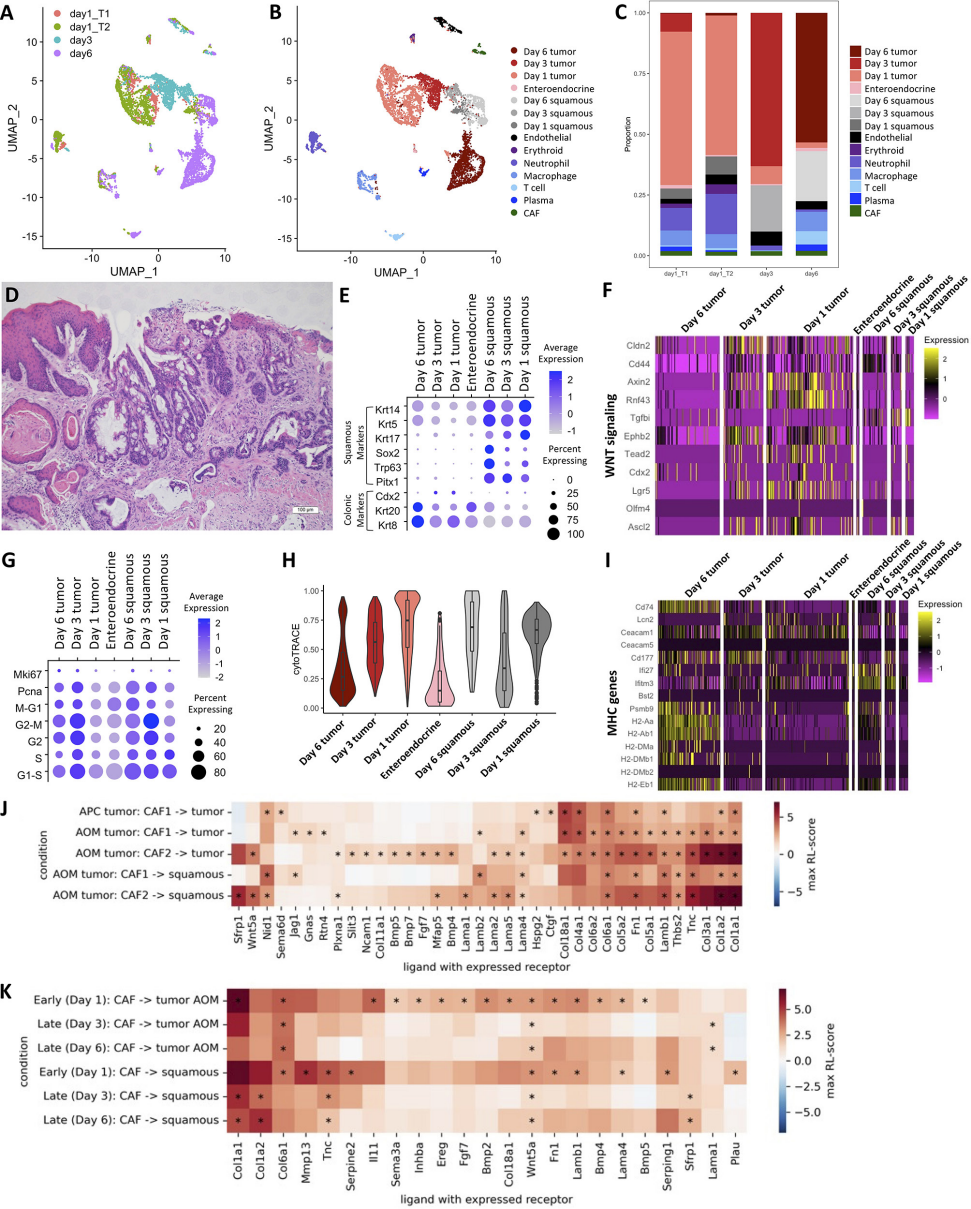
Changes in TME interactions within AOM/DSS tumors upon chemotherapy treatment. UMAP of scRNA-seq data from 5-FU and Irinotecan treated AOM/DSS tumor samples with timepoint overlay **(A)** and cell type clustering overlay **(B)**, by color. T1 and T2 indicate day 1 biological replicates (n = 2) that correspond to the “early treatment” timepoint, while day 3 and day6 are biological replicates (n =2 ) for the “late treatment” timepoint. **(C)** Bar graph of cell type proportions, broken down by sample type in timepoints of 5-FU and Irinotecan treated AOM/DSS tumor scRNA-seq data. **(D)** Representative H&E image of 5-FU and Irinotecan treated AOM/DSS tumors. Scale bar = 100 µm. **(E)** Dot plot showing colonic or squamous identity genes for epithelial cell clusters of 5-FU and Irinotecan treated AOM/DSS tumor scRNA-seq data timepoints. **(F)** Heatmap of genes related to WNT signaling and colonic stem cells. Columns represent single cells, arranged by epithelial cell type clusters in 5-FU and Irinotecan treated AOM/DSS tumor scRNA-seq data timepoints. **(G)** Dot plot showing cell cycle meta-genes for each cell cycle phase in epithelial cell clusters of 5-FU and Irinotecan treated AOM/DSS tumor scRNA-seq data timepoints. **(H)** CytoTRACE scores for epithelial cell types in 5-FU and Irinotecan treated AOM/DSS tumor scRNA-seq data timepoints. **(I)** Heatmap of select DEGs and marker genes related to AP. Columns represent single cells, arranged by epithelial cell type clusters in 5-FU and Irinotecan treated AOM/DSS tumor scRNA-seq data timepoints. **(J)** RL-scores for differentially expressed ligands in CAF cells with a receptor in squamous, AOM/DSS tumor, or APC tumor cells. **(K)** Ligands in CAF cells with a receptor in squamous or cancer cells early and late after treatment by 5-FU + Irinotecan. (*) indicates that both receptor and ligand are differentially expressed (adjusted p-value < 0.01) and that the log FC of the ligand is at least 0.5 for all samples of the same group.

We sought to determine how a chemotherapeutic regimen of Fluorouracil (5-FU) and Irinotecan affects the AOM/DSS TME. We treated AOM/DSS-induced tumor mice with a 5-FU and Irinotecan regimen for 1, 3, and 6 days and performed scRNA-seq on resulting tumors. To assess changes over the treatment timeline, we consider day 1 replicates as an “early treatment” timepoint, and days 3 and 6 as replicates for a “late treatment” timepoint. While chemotherapy is expected to induce cell death and tumor regression, our 5-FU/Irinotecan regimen coupled to the harvest time points did not result in reduction in tumor size, nor was there a reduction in cell numbers, which provides an opportunity to observe chemotherapy-induced changes within tumor cells. The tumor cells and squamous cells from days 1, 3, and 6 timepoints occupied different UMAP spaces, while stromal and immune cells from different timepoints largely overlapped (**Figures 5A, B**). Compared to the early treatment timepoint, we observed an expansion of squamous cells at late treatment timepoints (6 and 11% of total epithelial cells in early treatment, 21% and 27% of total epithelial cells in late treatment) (**Figure 5C**). However, differences in experimentalists and instrumentation prevent us from making comparisons of squamous cell percentages in the 5-FU/Irinotecan dataset to the untreated AOM/DSS dataset (**Figure 1F**). Histologically, we also observed squamous cell components located at the surface of the tumor, rather than underlying the tumor as in untreated AOM/DSS tumors, implicating a phenotypic transition (**Figure 5D**). Expansion of squamous cells was accompanied by increased expression of squamous identity transcription factors *Trp63* and *Sox2* (**Figure 5E**). Surprisingly, stem cell markers and associated WNT signaling genes, including *Lgr5*, decreased in tumor cells along the treatment timeline in a gradient-like manner (**Figure 5F**); this corresponded with increase in differentiated epithelial genes *Krt20* and *Krt8* (**Figure 5E**). Consistent with the loss of stemness upon treatment, tumor cells displayed an increase in cell cycle related genes at later timepoints and a decreased predicted stem potential, as determined by CytoTRACE score, whereas squamous cells increased proliferation genes and stem potential (**Figures 5G, H**). Using unsupervised differential gene expression analysis as well as examining selected marker genes, we observed an upregulation in antigen presentation (AP) genes in tumor cells from the day 6 timepoint, consistent with our previous finding of increased AP as a function of differentiation (**Figure 5I**) (26). Interestingly, squamous cells in day 3 and day 6 timepoints also upregulated interferon-induced genes *Ifi27* and *Ifitm3*, consistent with a change in the immune microenvironment. Together, we found that along a treatment timeline, 5-FU and Irinotecan treatment is associated with expansion of squamous cells and decreased stemness in tumor cells.

To further evaluate how tumor cells may be influenced by the TME, we analyzed differences in cell-cell communication of AOM/DSS tumors as compared to APC tumors, and moreover, we identified differences in cell-cell communication among early vs late 5-FU/Irinotecan treatment timepoints in AOM/DSS tumors. We made use of established receptor and ligand (R-L) interactions from a prior knowledge database and, similar to previous work, we quantified these interactions by a R-L score based on the receptor and ligand expression (76, 77). We searched for cell types expressing ligands that could bind receptors expressed in the tumor cells (**Figures S10A–D**). We observed that compared to all other cell types, CAF populations, particularly CAF2s, dominate interactions with tumor cells by providing ligands that are binding partners with receptors expressed on tumor cells (**Figure 5J**; **Figures S10A–D**). Many receptors expressed by tumor cells were also expressed by squamous cells, resulting in similar scores, and suggesting that they may experience similar signals from the TME. Consistent with the observed increase in WNT related genes in tumor cells (**Figures 4A**, **5F**), we saw high scores for WNT-related interactions (ligands *Sfrp1* and *Wnt5a* and receptors *Fzd6*, *Fzd7* and *Lrp6*) and significant BMP-related interactions (e.g. from *Bmp4* with *Bmpr1a)*. Notably, *Fgf7* is upregulated in acute response to injury and thought to serve a protective role in DSS treated mice, however, it is also known to increase cell proliferation and may inadvertently act as a growth factor for tumor cells in the AOM/DSS setting (78, 79). For squamous cells, there was a significant *Lama1-Rpsa* interaction, which is an interaction associated with metastasis as *Rpsa* enables tumors to penetrate laminin tissue (80). These interactions were largely mirrored in the 5-FU and Irinotecan treated cells, and the treatment appeared to reduce the amplitude of these signatures over time, possibly resulting in decreased stemness (**Figure 5K**).

## Discussion

We describe differences between the TMEs of AOM/DSS tumors and APC tumors, as models for pre-invasive cancers and pre-cancerous adenomas, respectively. Using single-cell and computational approaches, we identified and validated two CAF populations that are increased in AOM/DSS tumors, with CAF2 being uniquely present and the main driver of differences in cell-cell communication between the two colonic tumor models. CAF2-derived ligands interact with receptors expressed on AOM/DSS tumor cells to increase WNT signaling, increase growth factor signaling, and remodel the extracellular matrix. We also revealed a unique squamous cell component in many AOM/DSS, but not APC, tumors, which are found to increase upon a chemotherapeutic regimen of 5-FU and Irinotecan.

While there are many different types of CAFs with diverse functions, in general, increases in CAFs accompany cancer progression (81). In CRC in particular, the consensus molecular subtype 4 (CMS4) is characterized by mesenchymal signatures (82), with the majority contributed by the presence of CAFs (83–85). CMS4 CRCs are more advanced and have poor prognoses (86, 87). There are a diverse set of mechanisms by which CAFs promote CRC progression including WNT modulation (17, 41, 88), inhibition of BMP signaling (89), or inducing tumor-promoting inflammation (89). Our study identifies two populations of CAFs, one of which (CAF2) was not detected in APC adenoma by scRNA-seq, but was abundant in AOM/DSS tumors. CAF2s closely associate with tumor cells, which mirrors the major CAF dichotomy in pancreatic cancer, where myCAFs closely associates with neoplastic cells while iCAFs are located more distantly (49). However, the phenotypes of these colonic CAFs seem to be distinct from those of pancreatic cancers, as it is the CAF1s, which are located more distantly to epithelial cells, that have more myofibroblastic features and some iCAF gene expression such as *Il6* and *Cxcl1*, while CAF2s also express iCAF genes such as *Il11, Cxcl12* and other chemokines (40). The induction of immune-related genes in either CAF populations is specific to AOM/DSS tumors and not APC adenomas, suggesting that immune-regulating function of CAFs may only be activated in the context of DSS inflammation. CAF2s, which are closely associated with epithelium, parallel pericryptal fibroblasts or telocytes in the normal gut (90). These cells provide signals that support a WNT signaling niche to maintain stem cell renewal during normal homeostasis (46), although *Grem1* and *Wnt5a* seem to be expressed in distinct stromal populations in the non-neoplastic mouse colon (91–93). *Grem1/Wnt5a* co-expressing CAF2 may inhibit BMP while simultaneously stimulate WNT signaling, promoting stemness in more advanced tumors.

An unresolved question from this work is the origin of the squamous cells in AOM/DSS tumors. Similar histological findings of squamous cells in DSS colitis mouse models have been reported and were speculated to be a metaplastic event whereby the damaged colonic epithelium is re-epithelialized with stratified squamous epithelium (68). Squamous cell lesions of unknown origin have been observed in colonic polyps of CDX2-knockout mice, and likewise in SMAD3-depleted and SMAD3-knockout colonic tumors given DSS compared to DSS alone (94, 95). While squamous cell lesions could result from a simple re-epithelialization event, an alternative explanation is transdifferentiation of columnar colonic epithelial cells into stratified squamous epithelia, a process in reverse of normal colonic development. p63-positive pseudostratified embryonic epithelial cells from the endodermal lining of the primitive distal gut have been lineage traced to p63-negative adult colonic simple columnar epithelia (71). This supports the notion that some cancers, including colorectal cancer, may arise through embryonic subversion (96). In line with these observations, our data also showed an enrichment of fetal markers in the squamous tumor component absent from epithelial cells from both AOM/DSS and APC tumors, including genes expressed in primitive gut (*Trp63, Sox2, Pitx1)*, as well as metaplastic genes such as *Tacstd2.* We attempted to determine the origin of squamous lesions in AOM/DSS tumors using lineage tracing from *Lrig1*-expressing epithelial cells, since *Lrig1* is not expressed in the anal epithelium (75). We found rare lineage tracing events from *Lrig1*-expressing cells to a squamous lesion in an AOM/DSS tumor. While this result suggests the possibility of a transdifferentiation event, we cannot rule out sporadic, induced expression of *Lrig1* in the anal epithelium during re-epithelialization. We also observed AOM/DSS tumors, during a course of 5-FU and Irinotecan, expanded their squamous component. These cells exhibited more transcriptional similarity to tumor cells than those from untreated tumors, suggestive of transdifferentiation. However, this expansion could be due to the insensitivity of squamous cells to chemotherapy-induced cell death or drug-induced proliferation. Our L-R inference suggests that similar CAF-tumor interactions exist for CAF-squamous interactions. Moreover, we found that CAF2s lie in close proximity to not only AOM/DSS tumor cells, but also squamous lesions within tumors. The squamous transdifferentiation process has been shown to affect tumor cell interaction with CAFs in pancreatic cancer (69). We also found that differences in genetic backgrounds contribute profoundly to the development of squamous lesions, highlighting the complexity of factors that influence their development. Our findings implicate a role of squamous cells in colorectal tumorigenesis processes and provides insight into adenosquamous carcinomas in humans, a rare cancer type that is more aggressive and has a poorer prognosis when compared to adenocarcinoma, in part due to lack of mechanistic knowledge of their pathogenesis (97).

We also presented translationally relevant insights from our mouse studies. While checkpoint immunotherapies can result in long term responses, only a minority of cancer patients benefit (98). A positive immunotherapy response is largely dependent a favorable tumor microenvironment partly characterized by infiltration of adaptive immune cells (99–102). Some chemotherapies have been shown to stimulate the immune microenvironment, and thus, may boost the efficacy of immunotherapies when given as a combinatorial regimen. A major immunogenic mechanism is chemotherapy-induced immunogenic cell death, where tumor antigens and danger-associated molecular patterns are released into the microenvironment from a dying cell (103, 104). Our study proposes an additional mechanism, where a chemotherapy regimen of 5-FU and Irinotecan results in tumor cells with decreased WNT signaling, increased differentiation, and increased expression of AP machinery. MHC class I upregulation has been previously observed in a subcutaneous injection model of breast cancer under Irinotecan treatment (105), lending additional support to our observations. How this phenotype is achieved remains to be investigated, but a possibility may be that the chemotherapy kills highly proliferative stem cells while sparing less proliferative differentiated cells. Previous studies from our lab and others have demonstrated that tumor cell differentiation state controls antigen presentation and cytotoxic versus suppressive immunity (26, 106). However, cytotoxic T cells were not increased under 5-FU and Irinotecan despite increased expression of antigen presentation machinery, suggesting that additional immunosuppressive mechanisms may be activated. Indeed, previous studies show that Irinotecan treatment of AOM/DSS tumors blocks the apoptosis of MDSCs (107). Whereas chemotherapy regimens are non-targeted, further work in this area is required to discover therapeutic approaches to precisely establish and maintain a favorable immune microenvironment.

## Methods

### Mouse Experiments

All animal experiments were performed under protocols approved by the Vanderbilt University Animal Care and Use Committee and in accordance with NIH guidelines. Animals of C57BL/6J; 129 mixed or C57BL/6J backgrounds of the appropriate size by weight (6-12 weeks old) were used at the start of experiments. Mice were housed (2 to 5 per cage) in a specific pathogen-free environment under a standard 12-hour daylight cycle, and were fed a standard rodent Lab Chow and provided water ad libitum. Littermate controls of both sexes were used for experiments when possible.

For the APC tumor model, *Lrig1^CreERT2/+^;Apc^fl/+^* mice were induced as documented previously (18). For the AOM/DSS tumor model, mice were injected twice with 10 mg/kg AOM with one week apart. A week after the second injection, mice were fed 2% DSS for a week, and then again after 3 weeks of rest. For both models, tissues were harvested 12 weeks after the initial injection to initiate tumors, a timepoint where tumors are consistently present.

For lineage tracing studies, Lrig1^CreERT2/+;^Rosa26^LSL-EYFP/+^ mice were injected intraperitoneally (i.p.) for 3 consecutive days with 2.5 mg tamoxifen (Sigma-Aldrich; T5648) in corn oil. Mice were euthanized at the indicated timepoints for short and long-term lineage tracing.

For 5-FU and Irinotecan treatment, AOM/DSS tumors were first confirmed by colonoscopy after 12 weeks of induction. 50 mg/kg of Irinotecan hydrochloride (Millipore Sigma: 136572-09-3) was delivered intraperitoneally on day 1, followed by 50 mg/kg of 5-FU (Millipore Sigma: 51-21-8) on day 2, followed by 5 days of rest. Three cycles of this chemotherapy regimen were administered. Tissues were harvested according to timeline after the last cycle of treatment was given.

### Whole Tissue Single-Cell RNA-Sequencing and Initial Processing

Dissociation of colonic tumors was performed in a two-phase process. In the first stage, colon adenomas were dissected from the distal colon and washed in ice-cold PBS. The tumors were digested in DMEM containing 2 mg/mL collagenase type II at 37°C for 1 hour or until fragments had dispersed. The tumor tissue suspension was washed in ice-cold PBS and filtered through a 40 μm filter. Tumor epithelial crypts retained by the filter were collected and resuspended in PBS while the flow-through was discarded. The tumor epithelial fraction was filtered again through a 100 μm filter to remove undigested fragments, and the flow-through was collected. In the second stage, isolated tumor epithelial crypts were further digested into single cells for encapsulation similar to above. Normal colonic tissues were digested according to a previous protocol (108). Single-cell encapsulation, library preparation, and sequencing were performed as previous (109). Raw sequencing data were aligned using dropEst to mouse GRCm38.85 resulting in count matrices (110). After obtaining the count matrices, we combined the data for each sample and removed low-quality cells. We defined low-quality as any cell with either less than 500 genes detected or with greater 15% of counts mapping to mitochondrial genes. In total, we retained sequencing data of approximately 19,000 cells across all samples.

### Colonic Stroma Enrichment for Single-Cell RNA-Sequencing

Colon was isolated, flushed and washed briefly in DPBS (-ca/mg) with 20mM HEPES (DPBSH). Tissue was chelated at 37˚c for 20min with rotation in DPBSH with 5mM EDTA and 1mM DTT. After, the tissue was shaken in 10ml volumes of DPBSH to remove crypts. The remaining tissue (lamina propria and stroma) was then minced into ~2mm^2^ pieces and digested for 30min at 37˚C in DPBSH with 1mg/ml collagenase and dispase. Digest was then stopped by adding an equal volume of DPBSH with 5% FBS and 2.5mM EDTA. Tissue was triturated using a p1000 pipette and passed through a 70µm filter into a 50ml tube, with 10 additional mL of DPBSH to rinse. Cells were pelleted at 500g and resuspended in 5ml of 40% percol and underlayed with 5ml of 90% percol, then centrifuged at 500g for 10 minutes with no brakes to allow separation of cells. Layers at the top of the 40% percol and 40%/90% interface were washed 3 times in DPBS and loaded onto inDrop for encapsulation.

### Single-Cell RNA-Sequencing Data Analysis

Analysis of pre-processed murine scRNA-seq data was carried out in RStudio version 4.1.1 and Seurat version 4.0.4 (111). Functions use default arguments unless specified. Batch effects were minimal as seen from the intermixing of non-neoplastic cells, and therefore, no batch corrections were performed. After quality control and filtering steps, the following steps were performed for the following datasets: 1) APC tumor (n=2), AOM/DSS tumor (n=3), APC adjacent normal (n=2), and wildtype colon (n=2) samples (~13,000 total cells). 2) 5-FU and Irinotecan treated AOM/DSS tumor samples (n=2 for the “late treatment” timepoint from day 3 and 6 post-treatment, and n=2 for the “early treatment” timepoint from day 1 post-treatment, ~7,000 total cells). 3) Normal mouse colon enriched for fibroblasts (n=1), 1108 total cells, 465 of which are fibroblasts. A Seurat object was created using the CreateSeuratObject function and the number of genes, number of UMIs, and percent mitochondrial expression for all cells in the dataset were visualized. The NormalizeData, FindVariableFeatures (vst method with 2000 features), and ScaleData (using all genes) functions were used to normalize the data, find highly variable genes (HVGs), and scale and center the data. Principle component analysis (PCA) was performed using Seurat’s RunPCA function using only HVGs as features for dimension reduction. The jackstraw method (Seurat’s JackStraw function with 30 PCs) and the ElbowPlot function were used to determine the PCs to use for UMAP (Seurat’s RunUMAP function) visualization. DPC clustering was performed on the APC, AOM/DSS, APC adjacent, and wildtype dataset to determine cell types, with the exception of subclustering of the T cells, for which we used Seurat’s FindClusters function (112). For the 5-FU and Irinotecan-treated samples, we used Seurat’s FindClusters function as well as manual subclustering based on sample type. To determine the appropriate number of clusters in each sample, the clustering resolution parameter was adjusted, and differential expression analysis was performed to identify known cell types based on marker gene expression. We used Seurat’s FindMarkers function with standard parameters and log fold change thresholds above 2 to identify differentially expressed genes. Cell cycle analysis was performed as previously described (113). Sc-Unifrac was run with default parameters and using our clustering data, rather than the default hierarchical clustering, as previously described (23). The CytoTRACE web tool was used to calculate predicted stemness of single cells (74). Gene lists for metaplasia and damage, fetal and embryonic, and WNT signaling were used as previously described (26).

Analysis of human scRNA-seq data was performed in Python using scanpy, pandas, and numpy packages as previously described (26). Briefly, raw scRNA-seq counts were normalized by median library size, log-like transformed with Arcsinh, and Z- score standardized per gene. Cells annotated as mesenchymal cell types were subset from the Samsung Medical Center dataset (31). Similarly, cell annotated as mesenchymal from only microsatellite stable (MSS) tumor status samples were subset from the Broad Institute Human Tumor Atlas Network dataset (17). UMAP visualization was based on normalized gene counts for the two datasets. All UMAP plot coordinates for individual cells were generated with the *scanpy.tl.umap* function. The input to this function was the normalized dataset, their 40 principal components, and a KNN graph with k equal to the square root of the number of cells in the dataset. The *scanpy.pl.umap* function was used to visualize select gene marker expressions as an overlay onto UMAP coordinates.

### Immunofluorescence Imaging and MxIF

Colonic tissue was isolated, washed with 1X DPBS, spread longitudinally onto Whatman filter paper and fixed in 4% PFA (Thermo Scientific). Fixed tissues were washed with 1X DPBS, swiss-rolled, and stored in 70% EtOH until processing and paraffin embedding (formalin-fixed paraffin embedded, or “FFPE”), or were incubated in 30% sucrose in 1X PBS and embedded in cryo embedding media (“fixed frozen”). Tissues were sectioned at 5 or 7 um thick onto glass slides. FFPE tissue slides were processed for deparaffinization, rehydration, and antigen retrieval using citrate buffer (pH 6.0; Dako) for 20 minutes in a pressure cooker at 105°C followed by a 20-minute bench cool down. Endogenous background signal was reduced by incubating slides in 1% H_2_O_2_ (Sigma-Aldrich) for 10 minutes, before blocking for 30 minutes in 2.5% Normal Donkey Serum, 1% BSA in 1X DPBS prior to antibody staining. Primary antibodies against selected markers were incubated on the slides in a humidity chamber overnight, followed by three washes in PBS or dilute blocking buffer, and 1 hour incubation in Hoechst 33342 (Invitrogen), and compatible secondaries (1:500) conjugated to Invitrogen AlexaFluor-488, -555, or -647. Slides were washed in 1X DPBS or dilute blocking buffer, mounted in 50% glycerol/50% 1X PBS and imaged using a Zeiss Axio Imager M2 microscope with Axiovision digital imaging system (Zeiss; Jena GmBH). Multiplexed imaging using an immune cell-based antibody panel was performed by using a multiplex iterative staining and fluorescence-inactivation protocol, as previously described (47, 114), and imaged on an Olympus X81 inverted microscope with a motorized stage or Cytell Slide Imaging System (GE Healthcare) at 20X magnification. For histological analysis, slides were processed and stained for hematoxylin and eosin using standard approaches.

### Quantification of CAF Subtypes in MxIF Images

Stitched images were generated of tumor sections stained for nuclei with Hoechst and with antibodies against VIM, SMA, PDGFRα, TNC, and β-catenin. For each antibody target, thresholding was used to generate a binary mask. Tumor areas were outlined by identifying areas with strong β-catenin signal. Within tumor areas, CAF1, CAF2, and β-catenin binary masks were generated. CAF1 area was determined by overlaying binary masks of VIM and SMA signal, while CAF2 area was determined by overlaying binary masks of PDGFRα and TNC. CAF1 and CAF2 abundance was determined by normalizing the area of positive signal in CAF1 and CAF2 binary masks, individually, to the area of positive signal in the β-catenin binary mask. Example quantification is shown in **Figure S6**, and all MxIF data and masks are shown in **Figure S7** for APC tumors and **Figure S8** for AOM/DSS tumors. Student’s t-test was performed to determine statistical significance between 2 groups.

### Computation of Receptor Ligand Interaction Scores

In similar vein to previous work (76), the analysis aimed to identify receptor-ligand (RL) interactions using prior knowledge of their interactions and differential expression in cell-type pairs of interest. Here quantified as a log transformed product of fold changes (FC), RL-score = log2(FC_Ligand_×FC_Receptor)._ The interaction was taken to be significant if both the receptor and ligand were differentially expressed compared to background (see below). A multiple hypothesis corrected p-value threshold of 0.01 was used and results were also filtered for effect size using a cutoff of 0.5 log2 FC for ligands and cutoff of 0 (i.e. positive) for receptors. These results were further restricted to only include receptors and ligands where the filtering requirements were met for all samples within a group (n=3 for AOM/DSS, n=2 for APC and n=2 for early and late treatment by 5-FU + Irinotecan). A prior knowledge graph of receptor-ligand interactions was extracted from an online database (OmniPath) (77). The RL interactions were subset to ones with known mode of action and directionality. From this, lists of ligands and receptors were compiled and their differential gene expression was calculated. Gene expression was normalized on a cell by cell basis using the default method in the r-package Seurat (115), i.e. dividing the counts for each cell by the sum of counts, multiplying by 10000, and taking the log2(x+1) transformation. Differential gene expression and fold changes for different cell types was calculated on a sample-by-sample basis by comparing the gene expression of cells from one cell type and sample at a time against the background expression among all other cell types and samples. Differential gene expression was calculated with the FindMarkers function in Seurat using the MAST method (116) with filtering for fold change and percent gene expression set to 0. After computing p-values for all selected genes in all cell-types, multiple hypothesis testing adjusted p-values were calculated using the Benjamini & Hochberg method.

## Data Availability Statement

The datasets presented in this study can be found in online repositories. The names of the repository/repositories and accession number(s) can be found below: (https://www.ncbi.nlm.nih.gov/, GSE134255, GSE199999).

## Ethics Statement

The animal study was reviewed and approved by Vanderbilt University Animal Care and Use Committee.

## Author Contributions

PV, RC, DL, and KL study design and conception. PV, HN, AS, JR, JW, and WL experiment conduction. PV, AN, MK, ZC data analysis and statistical analysis. EM, BJ, MW, and JR technical consultation. KL and PV writing manuscript. All authors have approved the final manuscript.

## Funding

This work is funded by U01CA215798 to DL, R01DK103831 to KL, F31DK127687 to PV, R35CA197570 and P50CA236733 to RC, Vetenskapsrådet 2019-06349 to AN, T32HD007502 in support of PV, and P30CA068485 for cores used. RC acknowledges the generous support of the Nicholas Tierney GI Cancer Memorial Fund. The funders played no role in the research conducted.

## Conflict of Interest

The authors declare that the research was conducted in the absence of any commercial or financial relationships that could be construed as a potential conflict of interest.

## Publisher’s Note

All claims expressed in this article are solely those of the authors and do not necessarily represent those of their affiliated organizations, or those of the publisher, the editors and the reviewers. Any product that may be evaluated in this article, or claim that may be made by its manufacturer, is not guaranteed or endorsed by the publisher.
