# Supplementary figures and images for: Cancer-Associated Fibroblasts and Squamous Epithelial Cells Constitute a Unique Microenvironment in a Mouse Model of Inflammation-Induced Colon Cancer
Source: Front Oncol. 2022 May 4;12:878920. doi: 10.3389/fonc.2022.878920 (PMC9114773; doi:10.3389/fonc.2022.878920)

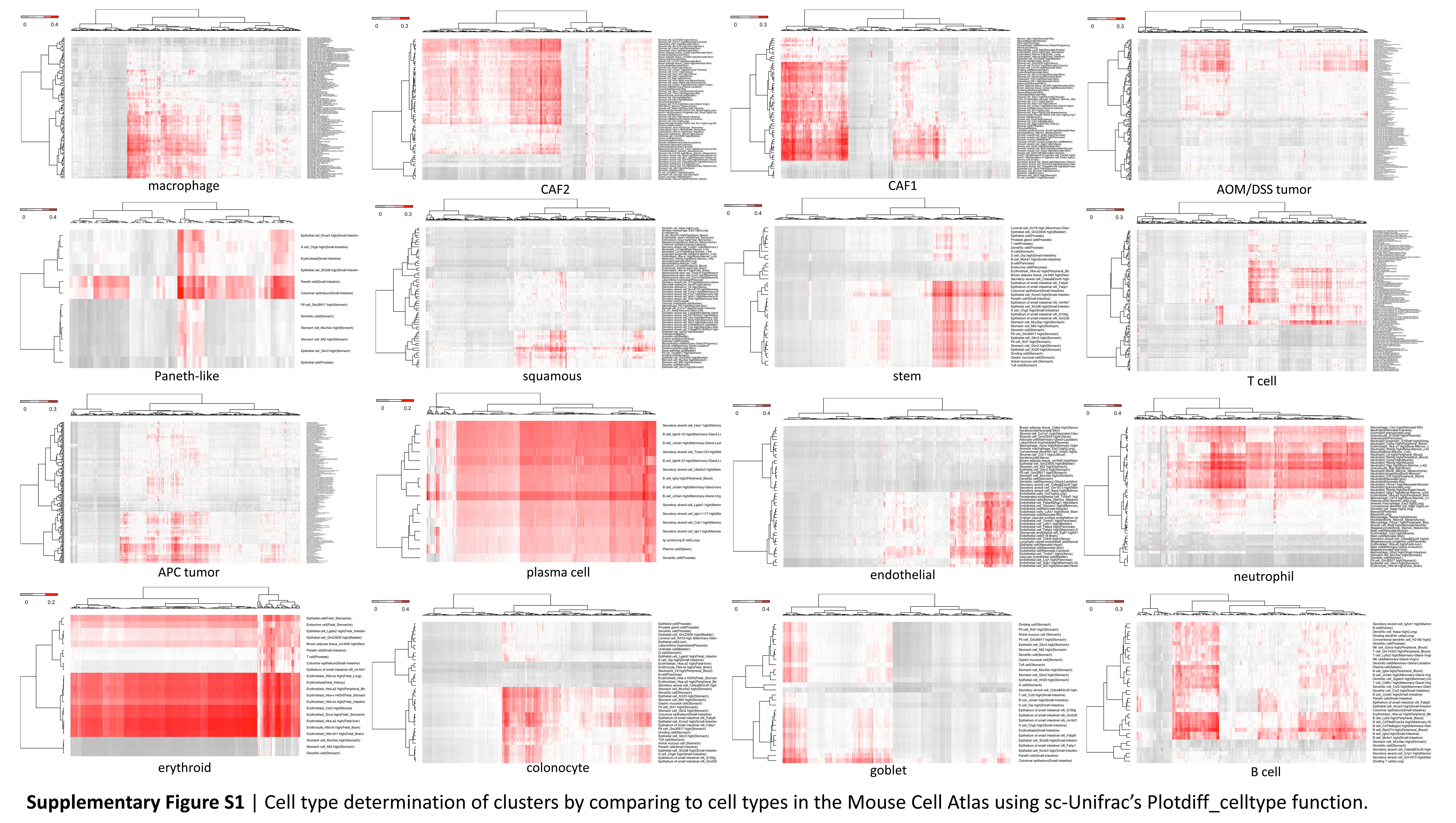

Supplement: Supplementary file 1 [file Image_1.jpg]

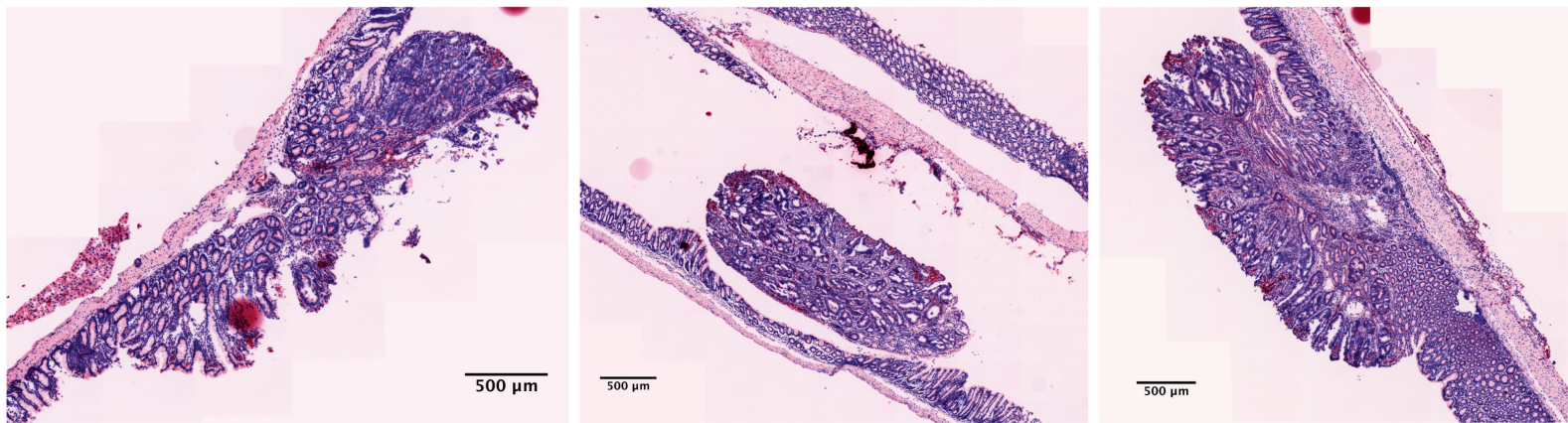

**Supplementary Figure S4** | Stitched virtual H&E images of APC tumors.

Supplement: Supplementary file 4 [file Image_4.pdf]

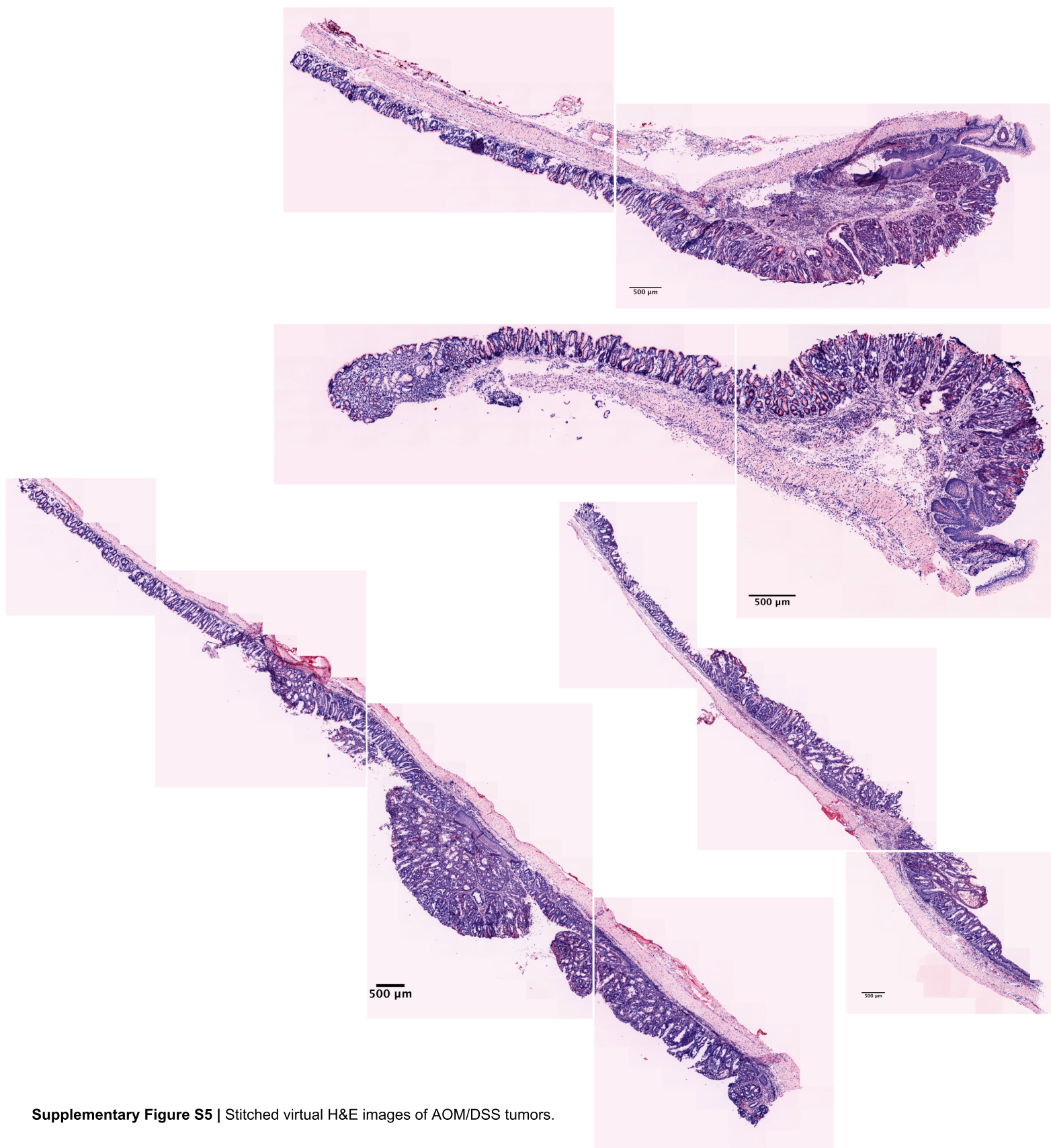

**Supplementary Figure S5** | Stitched virtual H&E images of AOM/DSS tumors.

Supplement: Supplementary file 5 [file Image_5.pdf]

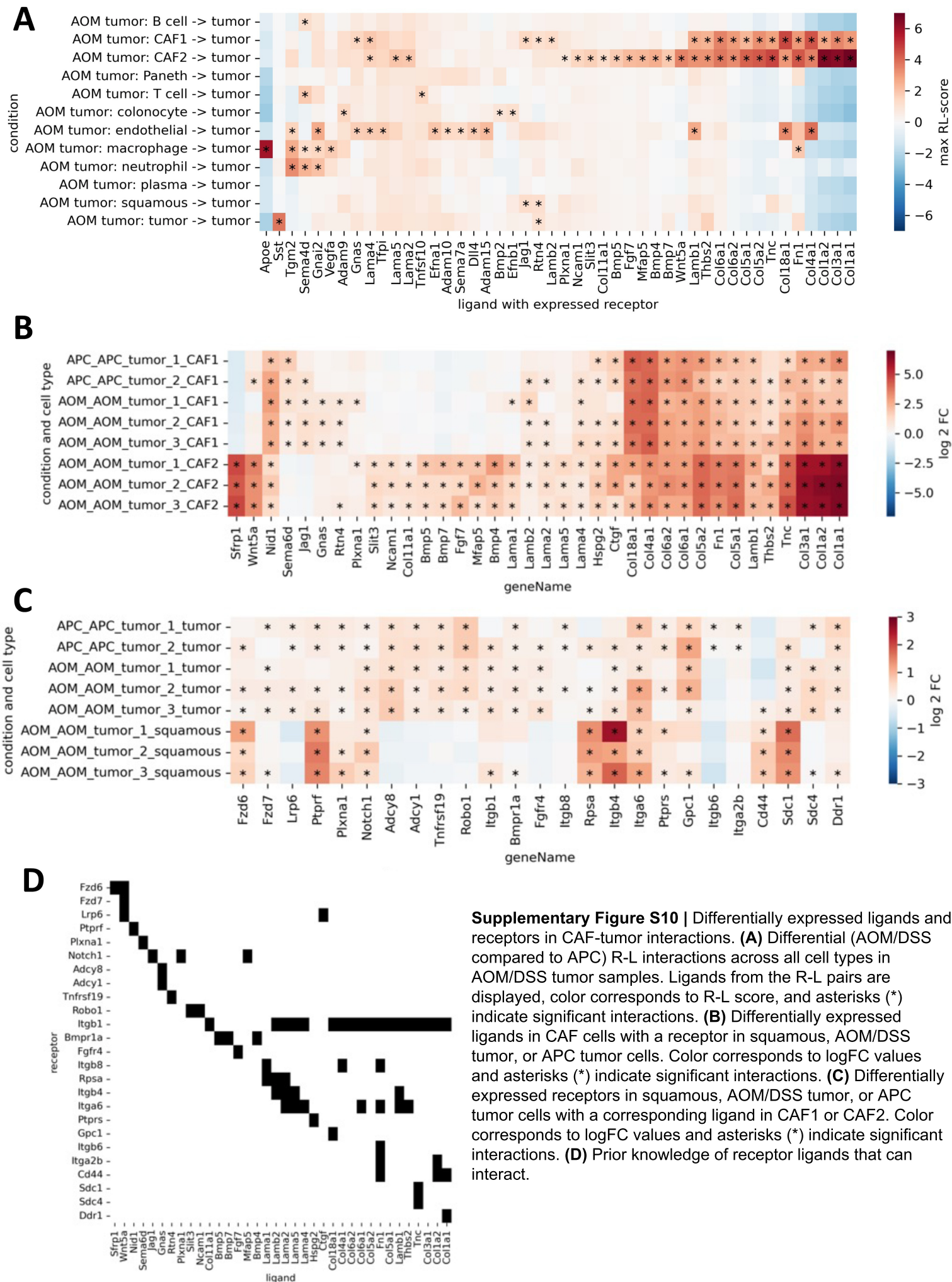

Supplement: Supplementary file 10 [file Image_10.pdf]
