# Supplementary material for: Cancer-Associated Fibroblasts and Squamous Epithelial Cells Constitute a Unique Microenvironment in a Mouse Model of Inflammation-Induced Colon Cancer
Source: Front Oncol. 2022 May 4;12:878920. doi: 10.3389/fonc.2022.878920 (PMC9114773; doi:10.3389/fonc.2022.878920)
Supplement: Supplementary file 3 [file Image_3.pdf]

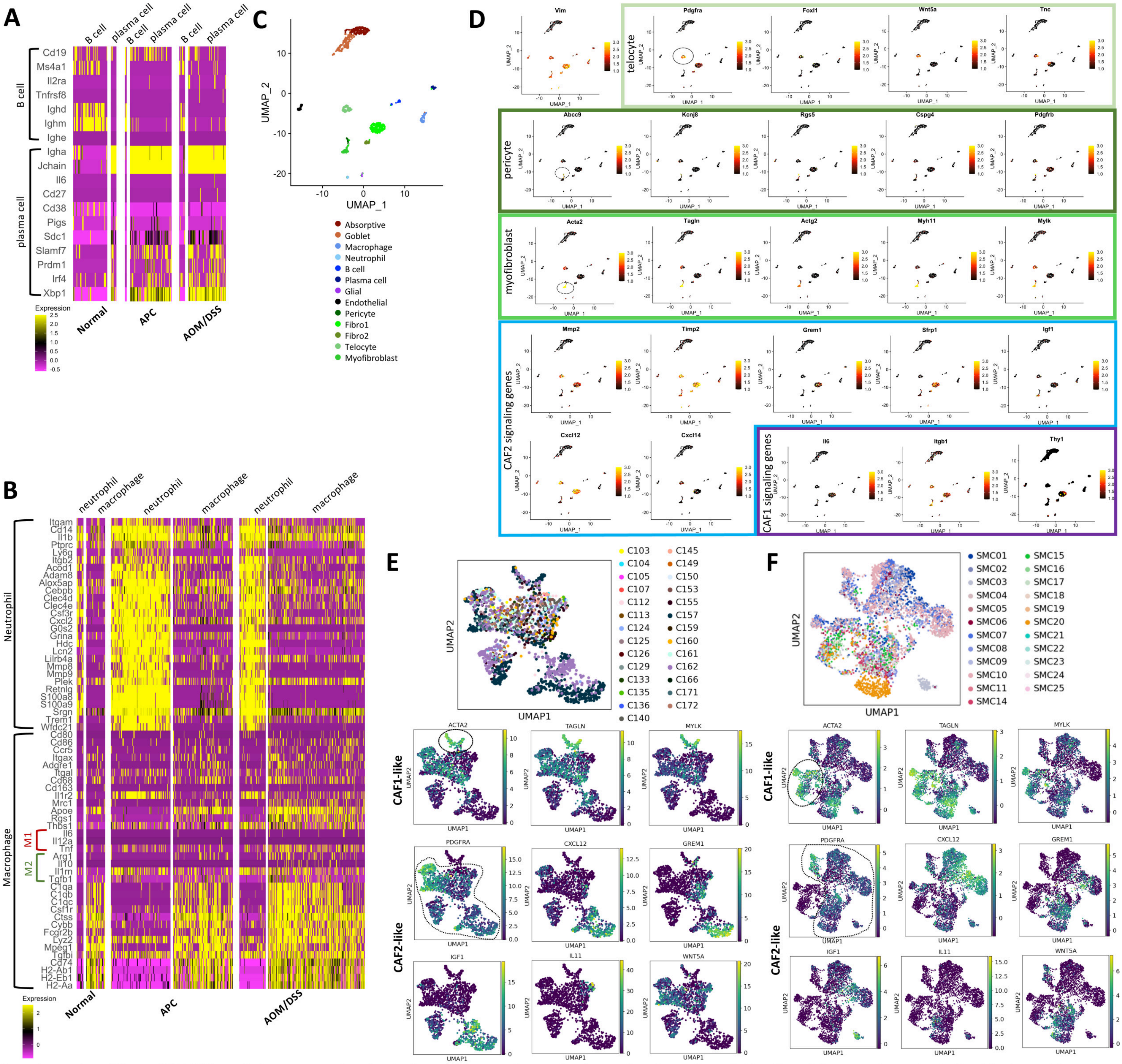

Supplementary Figure S3 | Differentially expressed and select marker genes for murine tumor B cells and myeloid cells, wildtype murine colonic fibroblasts, and human CRC fibroblasts. Heatmaps displaying expression of **(A)** B cell and plasma cell markers, and **(B)** neutrophil and macrophage markers in scRNA-seq data from normal colonic, APC tumor, and AOM/DSS tumor samples. Rows correspond to individual genes and columns are individual cells, arranged by cell type cluster. **(C)** UMAP of scRNA-seq data generated from a fibroblast-enriched colonic murine sample (n = 1) with cell type clustering overlay by color, or **(D)** selected gene expression overlay, grouped by boxes for markers of specific cell types. **(E)** UMAP of mesenchymal cells subset from human CRC scRNA-seq data from Broad Institute MSS samples with patient replicate overlay (top), CAF1 marker gene expression overlay (middle panels), and CAF2 marker gene expression overlay (bottom panels). Sketched circles indicate the CAF1-like or CAF2-like sub-cluster. n = 27 specimens. **(F)** UMAP of mesenchymal cells subset from human CRC scRNA-seq data from Samsung Medical Center samples with patient replicate overlay (top), CAF1 marker gene expression overlay (middle panels), and CAF2 marker gene expression overlay (bottom panels). Sketched circles indicate the CAF1-like or CAF2-like sub-cluster. n = 23 specimens.
