# Supplementary material for: Cancer-Associated Fibroblasts and Squamous Epithelial Cells Constitute a Unique Microenvironment in a Mouse Model of Inflammation-Induced Colon Cancer
Source: Front Oncol. 2022 May 4;12:878920. doi: 10.3389/fonc.2022.878920 (PMC9114773; doi:10.3389/fonc.2022.878920)
Supplement: Supplementary file 6 [file Image_6.pdf]

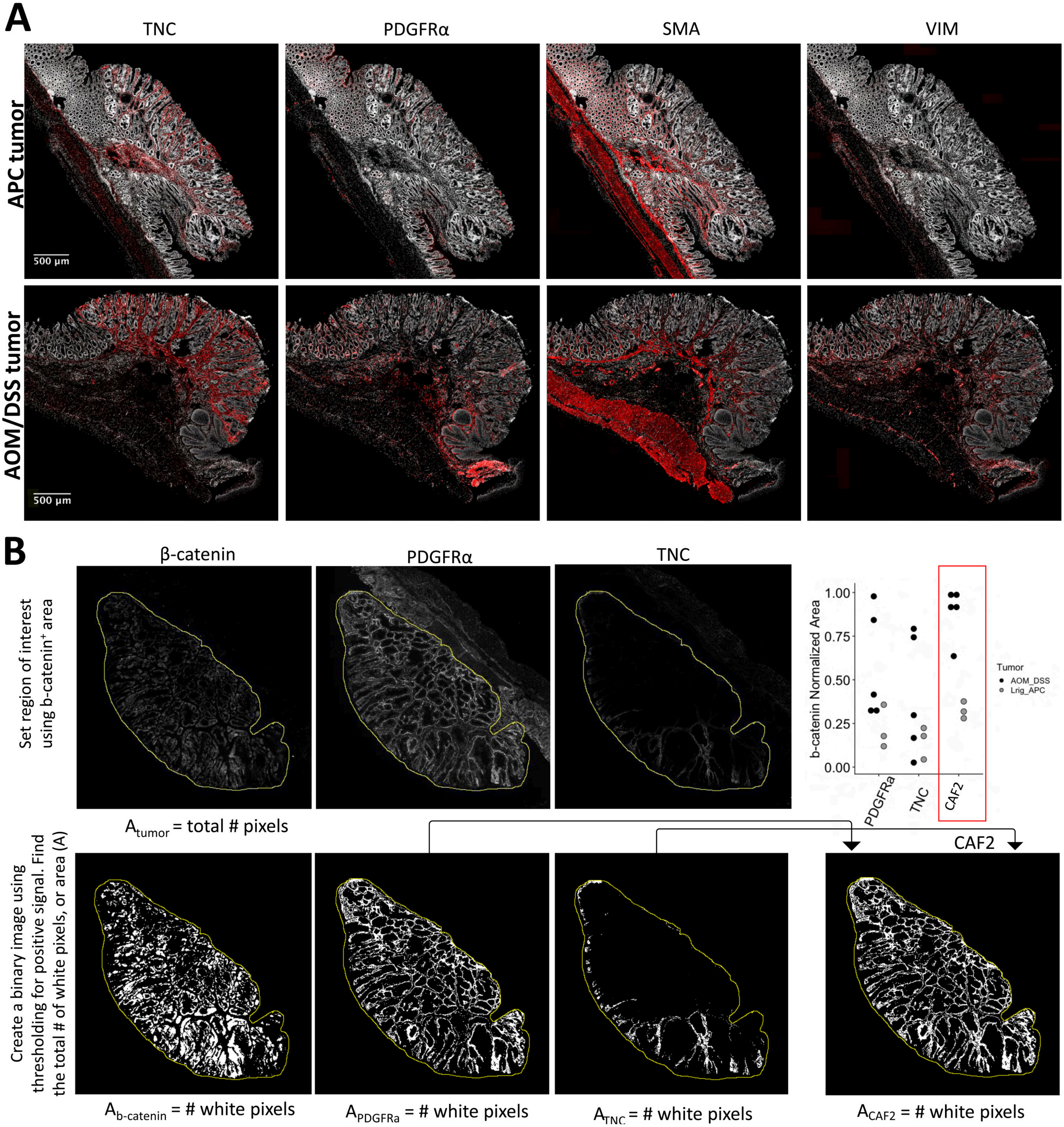


**Supplementary Figure S6 |** MxIF CAF markers and quantification method. **(A)** Single CAF markers with nuclei counterstain (white) from MxIF images in Figure 3A. **(B)** Workflow for MxIF quantification, using CAF2 quantification as an example.
