# Supplementary material for: Cancer-Associated Fibroblasts and Squamous Epithelial Cells Constitute a Unique Microenvironment in a Mouse Model of Inflammation-Induced Colon Cancer
Source: Front Oncol. 2022 May 4;12:878920. doi: 10.3389/fonc.2022.878920 (PMC9114773; doi:10.3389/fonc.2022.878920)
Supplement: Supplementary file 7 [file Image_7.pdf]

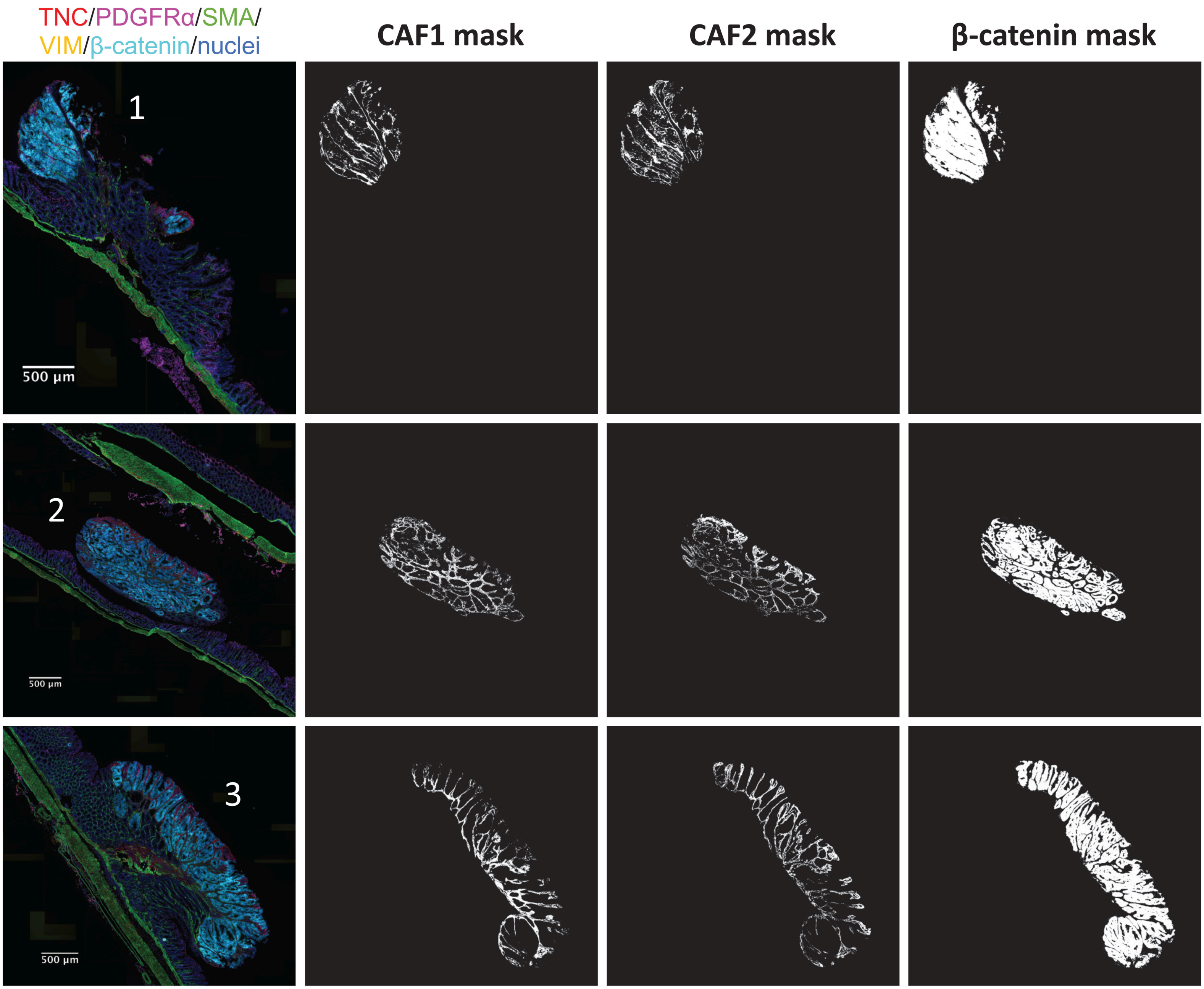

Supplementary Figure S7 | MxIF images and binary masks used for quantification of APC tumor CAF1 and CAF2 marker expression, normalized by β-catenin-expressing tumor area for n=3 tumors.
