# Supplementary material for: Cancer-Associated Fibroblasts and Squamous Epithelial Cells Constitute a Unique Microenvironment in a Mouse Model of Inflammation-Induced Colon Cancer
Source: Front Oncol. 2022 May 4;12:878920. doi: 10.3389/fonc.2022.878920 (PMC9114773; doi:10.3389/fonc.2022.878920)
Supplement: Supplementary file 8 [file Image_8.pdf]

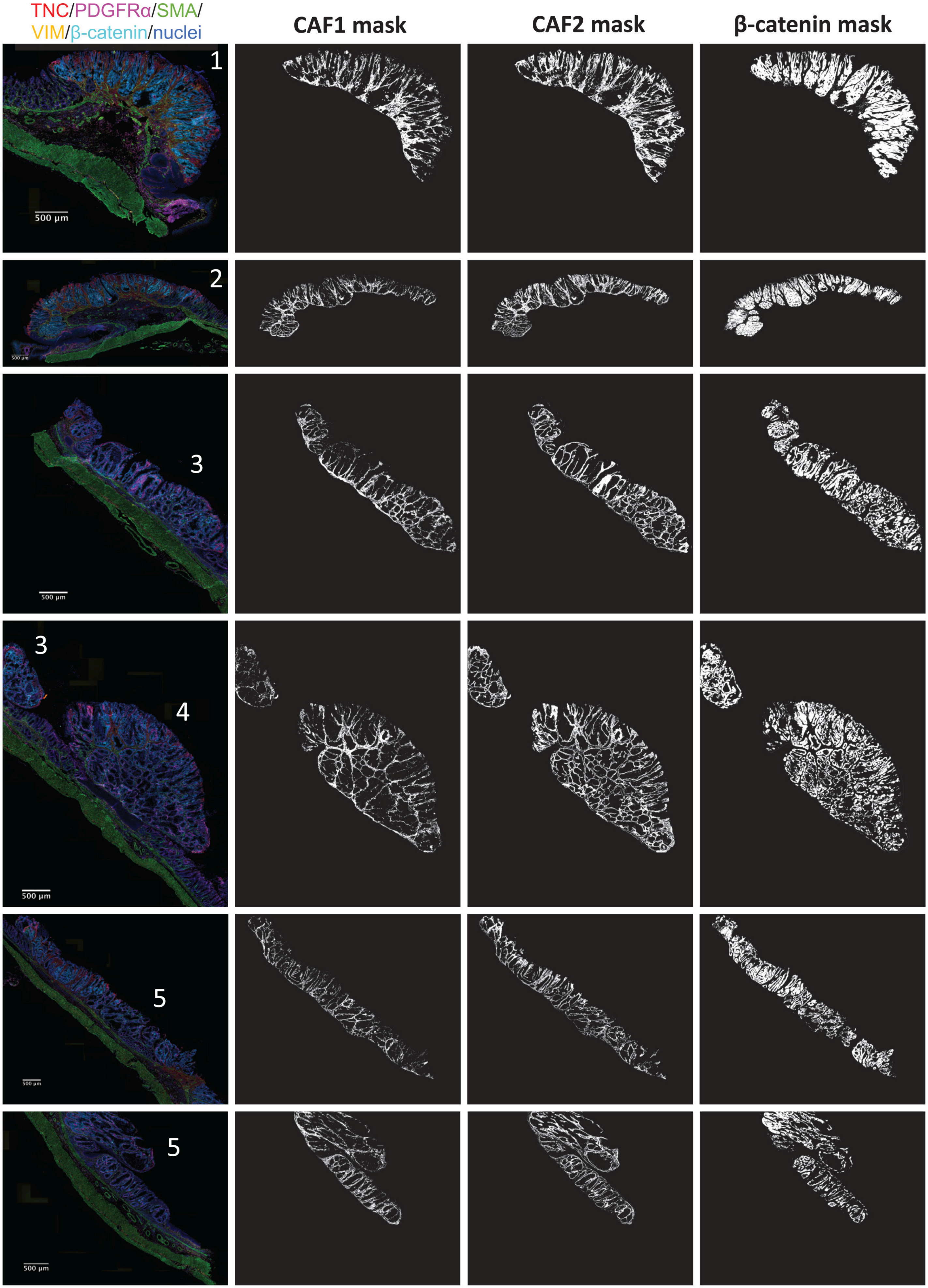


**Supplementary Figure S8 |** MxIF images and binary masks used for quantification of AOM/DSS tumor CAF1 and CAF2 marker expression, normalized by β-catenin-expressing tumor area for n=5 tumors. Integers 1-5 mark distinct tumors, where tumor "3" is repeated because the tumor spanned across two stitched images. However, the quantification was performed by considering the pixel areas of the two regions for tumor "3".
