# Supplementary material for: Cancer-Associated Fibroblasts and Squamous Epithelial Cells Constitute a Unique Microenvironment in a Mouse Model of Inflammation-Induced Colon Cancer
Source: Front Oncol. 2022 May 4;12:878920. doi: 10.3389/fonc.2022.878920 (PMC9114773; doi:10.3389/fonc.2022.878920)
Supplement: Supplementary file 9 [file Image_9.pdf]

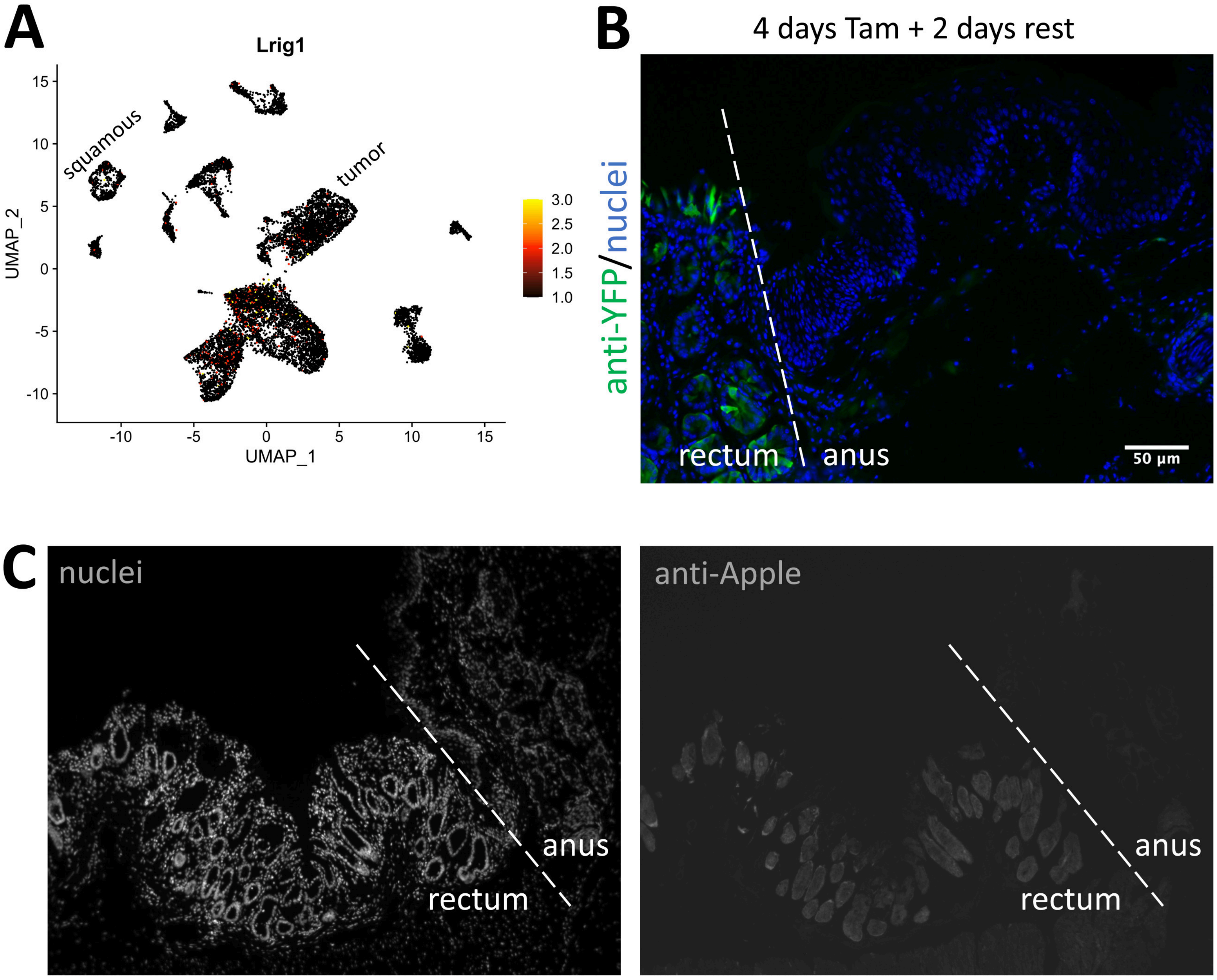

Supplementary Figure S9 | Lrig1 is not expressed in squamous cells. **(A)** Lrig1 expression in scRNA-seq data of wildtype, APC adjacent, APC tumor, and AOM/DSS tumor samples displayed as a UMAP overlay. Squamous and tumor cell clusters are labeled. **(B)** Representative IF image from lineage traced Lrig1CreERT2/+; RosaYFP/+ mice. YFP signal was visualized with an anti-YFP antibody, and nuclei are stained with Hoechst. **(C)** Representative IF image from Lrig1Apple/+ reporter mice. Apple signal was visualized with an anti-Apple antibody (right), and nuclei are stained with Hoechst (left). For both **(B)** and **(C)**, colon and anus were collected, and the anorectal junction is depicted by the dashed line. Magnification is the same for all images.
