# Supplementary material for: Cancer-Associated Fibroblasts and Squamous Epithelial Cells Constitute a Unique Microenvironment in a Mouse Model of Inflammation-Induced Colon Cancer
Source: Front Oncol. 2022 May 4;12:878920. doi: 10.3389/fonc.2022.878920 (PMC9114773; doi:10.3389/fonc.2022.878920)
Supplement: Supplementary file 2 [file Image_2.pdf]

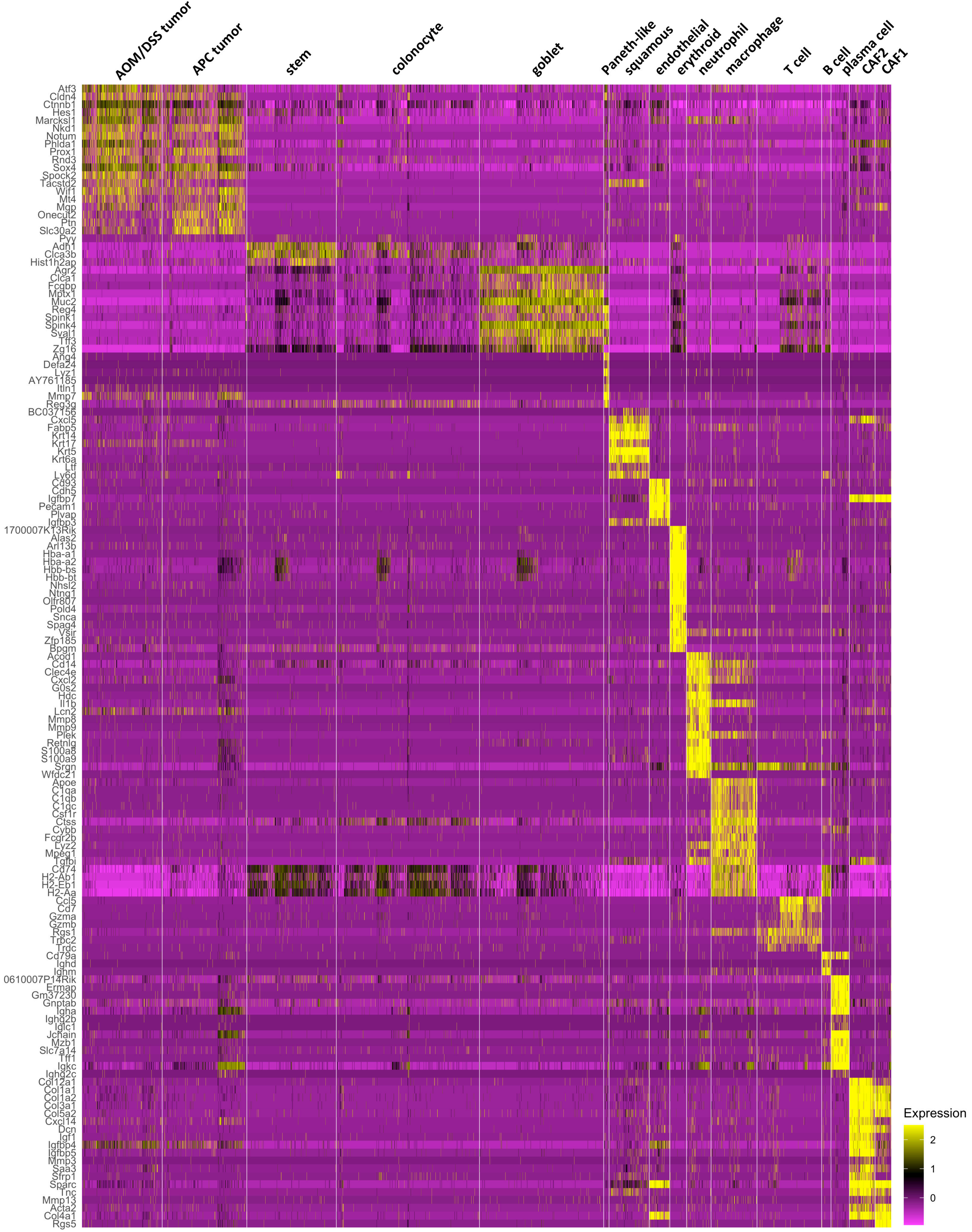


**Supplementary Figure S2 |** Heatmap displaying top differentially expressed genes in all clusters in scRNA-seq data from normal colonic, APC tumor, and AOM/DSS tumor samples. Rows correspond to individual genes and columns are individual cells, arranged by epithelial cell type clusters.
